# Monovalent manganese based anodes and co-solvent electrolyte for stable low-cost high-rate sodium-ion batteries

**DOI:** 10.1038/s41467-018-03257-1

**Published:** 2018-02-28

**Authors:** Ali Firouzi, Ruimin Qiao, Shahrokh Motallebi, Christian W. Valencia, Hannah S. Israel, Mai Fujimoto, L. Andrew Wray, Yi-De Chuang, Wanli Yang, Colin D. Wessells

**Affiliations:** 1 Alveo Energy, Palo Alto, CA 94303 USA; 2 Advanced Light Source, Lawrence Berkeley National Laboratory, Berkeley, CA 94720 USA; 3 Department of Physics, New York University, New York,, NY 10003 USA

## Abstract

The demand of sustainable power supply requires high-performance cost-effective energy storage technologies. Here we report a high-rate long-life low-cost sodium-ion battery full-cell system by innovating both the anode and the electrolyte. The redox couple of manganese(I/II) in Prussian blue analogs enables a high-rate and stable anode. Soft X-ray absorption spectroscopy and resonant inelastic X-ray scattering provide direct evidence suggesting the existence of monovalent manganese in the charged anode. There is a strong hybridization between cyano ligands and manganese-*3d* states, which benefits the electronic property for improving rate performance. Additionally, we employ an organic–aqueous cosolvent electrolyte to solve the long-standing solubility issue of Prussian blue analogs. A full-cell sodium-ion battery with low-cost Prussian blue analogs in both electrodes and co-solvent electrolyte retains 95% of its initial discharge capacity after 1000 cycles at 1C and 95% depth of discharge. The revealed manganese(I/II) redox couple inspires conceptual innovations of batteries based on atypical oxidation states.

## Abstract (web summary)

Sodium ion batteries offer more cost-effective storage than lithium and could be used for grid-scale energy storage. Here, the authors demonstrate a full cell based on a MnHCMn anode and an organic-aqueous cosolvent electrolyte. X-ray spectroscopy evidence further suggests the presence of Mn(I).

## Introduction

The widespread deployment of volatile renewable energy sources, such as solar and wind power, destabilizes the electric grid because the conventional power generation system based on fossil fuels cannot ramp quickly to balance the power variations from the renewable sources^1,2^. As a result, some jurisdictions now mandate the installation of energy storage on the grid where the renewables are prevalent (see J. Intrator et al., 2020 Strategic analysis of energy storage in California, 2011). Bulk stationary storage technologies such as pumped hydropower and compressed air energy storage are limited by the paucity of practical locations, high capital costs, and low energy efficiency^1,2^. Energy storage in the form of batteries could rapidly respond to such grid-level volatility. Unfortunately, the cost of most existing technologies, including lithium-ion, molten sodium, and redox flow batteries, is significant, which so far hinders their grid-scale applications^3^, US DOE report: Impact of Wind and Solar on the Value of Energy Storage, 2013). The emerging storage technologies such as aqueous sodium systems offer low cost for long-duration storage but do not provide the rate capability that is needed to balance the volatile power generation. In particular, it remains a critical challenge to develop a stable negative electrode (anode) for high-rate Na-ion battery system^4,5^.

**Fig. 1:**
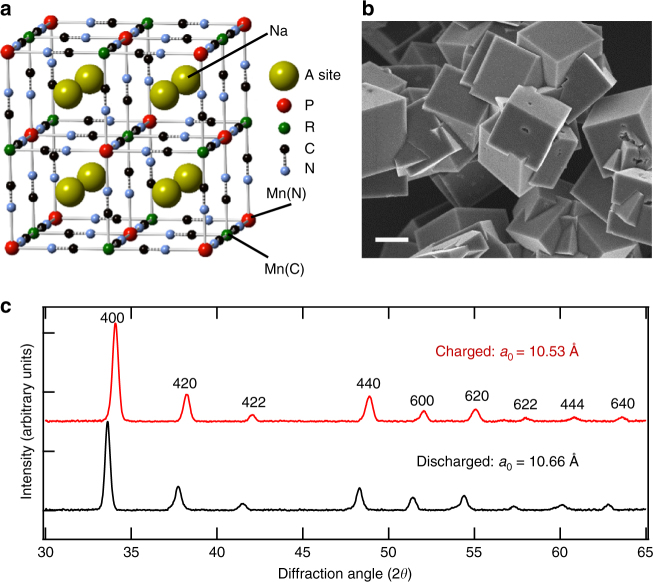
Structure of Mn-based PBA, MnHCMn, materials. **a** The unit cell of PBAs, in which transition-metal cations are octahedrally coordinated to C-N ligands in a cubic framework. The interstitial A sites store the alkali cations that are electrochemically intercalated and the zeolitic water. **b** A scanning electron micrograph of the as-synthesized MnHCMn material. Scale bar is 1 µm. **c** The powder X-ray diffraction spectra of a pristine (discharged) and charged MnHCMn electrodes. The cubic PBA structure is maintained throughout the electrochemical process

Prussian blue analog (PBA) batteries have received increasing attention because of the high rate, long cycle life, and low cost of PBA-based electrodes^6–11^. The general chemical formula for these materials is A_x_P_y_[R(CN)_6_]_z_·nH_2_O, where A is an alkali cation and P and R are transition metal (TM) cations. The face-centered cubic structure of PBA is composed of an open framework of octahedral hexacyanometalate complexes bonded to nitrogen-coordinated TMs (Fig. 1a). This framework contains large interstices (A sites) and channels that allow rapid intercalation of hydrated alkali cations such as sodium with near-zero strain. Therefore, PBA materials inherently feature long cycle life and high-rate capabilities, as have been exploited for both electrochromic and energy storage applications. Recently, the slurry-based composite electrode was modified to include PBA-active materials. PBAs are thus employed for inexpensive aqueous-electrolyte cells, as well as for high-voltage organic electrolyte sodium-ion cells^6–11^.

Although with their inherent high-rate and low-cost features, two technical challenges hinder the employment of PBAs for Na-ion batteries. First, the high potential (vs. standard hydrogen electrode (SHE)) from the typical transition-metal redox reaction in PBA materials suggests their employment as cathode in Na-ion batteries, as in previous reports^6–11^. However, a high-performance low-cost anode with a matching capacity for Na-ion batteries is still under scrutiny^4,5^. Especially, the commonly used activated carbon for Na-ion batteries has limited capacity of only 30 mAh g^–1^ at 1C with a relatively high cost^12^. Second, The solubility of PBA materials in water leads to an impractically short “calendar life” of a battery with PBA and aqueous electrolyte^6,13^. In anhydrous electrolytes, the kinetics for electrochemical cycling of PBAs is considerably slower than in water, decreasing the utility of these cells for high-rate applications^8,10^.

In this work, we tackle both of the aforementioned challenges by revealing the novel Mn(II/I) redox couple in a new cubic-phase manganese hexacyanomanganate (MnHCMn) for anode applications and by innovating a Na-ion organic–aqueous cosolvent electrolyte system. Scientifically, we provide the first conclusive evidence of the a Mn(I) state, which is observed in our MnHCMn anode at charged (reduced) state through soft X-ray absorption spectroscopy (sXAS) and resonant inelastic X-ray scattering (RIXS). Technically, we demonstrate a Na-ion battery full cell with superior rate and cycling performance. Our full cells with the copper hexacyanoferrate (CuHCF) cathode^6^ and MnHCMn(II/I) anode have an average cell voltage of 1.55 V, which is practical in grid-scale storage systems. Our organic–aqueous cosolvent electrolyte successfully solves the PBA dissolution issue. This full cell delivers 90% of its total energy during a 5-min discharge and thousands of deep discharge cycles. All components of our full Na-ion battery cells are inexpensive commodity materials. The cell fabrication is compatible with the existing Li-ion manufacturing processes without dry room requirements. The levelized cost of energy (LCOE) analysis shows that the projected LCOE of our all-PBA Na-ion battery full cell ($0.02 per kWh per cycle, Supplementary Notes 1 and 2) surpasses the aggressive US Department of Energy target for grid-scale energy storage (see US Department of Energy ARPA-E FOA document, 2010^14^).

## Results

### Material characterizations

The MnHCMn anode material is synthesized by the addition of aqueous sodium cyanide to aqueous manganese acetate. The rate of precipitation of MnHCMn from concentrated solutions is considerably slower than that of CuHCF, which results in large, sharply faceted cubic particles about 2 µm in size, as seen in the scanning electron microscope (SEM) image (Fig. 1b). X-ray diffraction (XRD) shows that the MnHCMn here has the cubic PBA structure (*a*_0_ = 10.66 Å) (Fig. 1c). The composition of the MnHCMn was determined using inductively coupled plasma optical emission spectroscopy (ICP-OES) (ratio of Na to Mn) and Carbon, Hydrogen, and Nitrogen (ratio of C/N to Mn) analysis (Intertek) to be Na_1.24_Mn[Mn(CN)_6_]_0.81_·2.1H_2_O. The 13% wt. water content of MnHCMn was confirmed using thermogravimetric mass spectroscopy (TGA-MS) (Supplementary Fig. 1). We note that the balanced valence of Mn is 2+ in this formula. In particular, previously reported MnHCMn was synthesized from a sodium cyanide precursor, which leads to the monoclinic and rhombohedral structures with low vacancy^15^. Our materials feature a high HCMn vacancy concentration of 0.19, which contrasts the low-vacancy (0.07) monoclinic phase^10^. We note that the same cubic structured high-vacancy Mn(II)HCMn(III) was previously reported by Pasta et al^7^. Besides the differences on the morphology and the pristine material, i.e., Mn(II)HCMn(II) here, our synthesis is based on inexpensive NaCN, and the material is free from K^+^ that could lead to potential increase or loss of capacity. The production of the cubic MnHCMn phase is due to the limited use of cyanide during the synthesis, compared with the excessive use in previous works. Our reaction solution contains a 2.57:1 CN:Mn ratio, which is consistent with the cubic phase produced in ref. ^7^ (CN:Mn = 3.3) but contrasts the monoclinic phases reported in ref. ^11,15^ (CN:Mn > 5).

The CuHCF cathode material was synthesized by precipitation from aqueous solution at room temperature by a method similar to those previously reported^6,7^. Characterization of CuHCF via SEM and powder XRD show a poorly faceted, nanoparticulate (100–200 nm) morphology with the phase-pure Prussian blue structure (*a*_0_ = 10.15 Å) (Supplementary Fig.s 2, 3). Analysis by ICP-OES and TGA-MS analysis shows a chemical formula of K_0.06_Cu[Fe(CN)_6_]_0.67_·3.8H_2_O (Supplementary Fig. 4).

### Electrochemical property of MnHCMn anodes

**Fig. 2:**
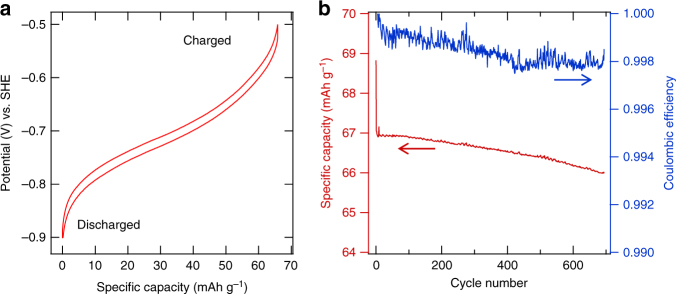
Half-cell electrochemical characterization of MnHCMn vs. SHE. **a** The reaction potential profile of the MnHCMn electrode vs. SHE at a 1C rate. Electrolyte is 1 M NaClO_4_, 90% MeCN, 10% water. **b** The cycle life and coulombic efficiency of the MnHCMn electrode during 1C–1C cycling between –0.5 and –0.9 V

**Fig. 3:**
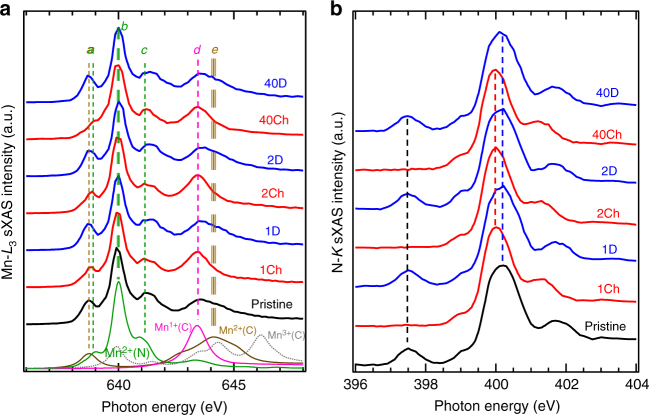
sXAS probe of the Mn^1+^/Mn^2+^ and the strong hybridization in MnHCMn electrodes. **a** Mn *L*_3_-edge sXAS spectra collected on a series of MnHCMn electrodes at the charged (reduced), marked with “Ch”, and discharged (oxidized), marked with “D”, states of the first, second, and fortieth cycles. Calculated spectra of Mn^II^(N), Mn^III^(C), Mn^II^ (C), and Mn^I^(C) are plotted at the bottom. The sXAS features are assigned to Mn with different oxidation states at different coordination sites (dashed lines). **b** The N *K*-edge sXAS spectra collected on the same series of MnHCMn electrodes. The red and blue dashed lines indicate the chemical potential shifts between the charged and discharged electrodes. The black dashed line indicates an unoccupied low-energy electron states from the hybridization of the NC ligands and Mn-3*d* states at the discharged (oxidized) states. This hybridization state disappears due to the electron filling during the electrochemical charging (reducing) process

We first characterize the electrochemical property (Fig. 2) and clarify the cycling mechanism (Fig. 3) of the cubic MnHCMn as a Na-ion battery anode through half-cell tests with SHE reference electrodes. The benefit of the used cosolvent electrolyte (1 M NaClO_4_, 90% acetonitrile (MeCN), 10% water) will be elaborated later in this work.

Figure 2a displays the cycling profile of MnHCMn vs. SHE between –0.5 and –0.9 V at 1C rate. The C-rates are defined with a reference capacity of 60 mAh g^–1^ (theoretical capacity 78 mAh g^–1^), which is typical for PBAs undergoing single electron transfer for redox reactions of the hexacyanometalate groups. The initial open circuit potential of the MnHCMn electrode in the half cell is approximately –0.1 V. The 1C cycling (CCCV charge, CV step to C/10) in the –0.5 to –0.9 V potential range yields a specific capacity of 67 mAh g^–1^ centered at –0.70 V, with 99.8% coulombic efficiency (Fig. 2b). This reaction corresponds to the intercalation of Na^+^ and the reduction of the HCMn groups:

1 $${\mathrm{Na}}_{{\mathrm{1}}{\mathrm{.24}}}{\mathrm{Mn}}\left[ {{\mathrm{Mn}}^{{\mathrm{II}}}\left( {{\mathrm{CN}}} \right)_{\mathrm{6}}} \right]_{{\mathrm{0}}{\mathrm{.81}}}{\mathrm{ + 0}}{\mathrm{.81\cdot }}\left( {{\mathrm{Na}}^{\mathrm{ + }}{\mathrm{ + e}}^{\mathrm{-}}} \right)\\ {\mathrm{ = Na}}_{{\mathrm{2}}{\mathrm{.05}}}{\mathrm{Mn}}\left[ {{\mathrm{Mn}}^{\mathrm{I}}\left( {{\mathrm{CN}}} \right)_{\mathrm{6}}} \right]_{{\mathrm{0}}{\mathrm{.81}}}$$

Two striking results can be seen from the electrochemical tests. First, the S-curve shape of the reaction profile indicates that the phase transformation is of a solid–solution-type reaction. Second, XRD characterization of electrodes at full charge and discharged showed that the structure remains cubic over its full state-of-charge (SOC) range with a small 1.2% decrease in lattice parameter during charging (Fig. 1c).

We note that both the solid–solution reaction and the stable structure of our cubic MnHCMn material is in contrast with the previous reports on the monoclinic MnHCMn phases, which show lower HCMn vacancy content, two-phase reactions, and a change in structure as the “A” site occupancy increases beyond 1 Na^+^ per interstice^10,11^. Retention of the cubic phase over the full SOC range of the reaction here is likely due to the high HCMn vacancy in our cubic material, which results in two Na^+^ per formula unit, i.e., full single occupancy of the interstitial A-sites (Fig. 1a).

Figure 2b shows the long-term 1C half-cell cycling of MnHCMn vs. SHE between –0.5 and –0.9 V. A linear loss rate of 18 ± 5 p.p.m. per cycle (*N* = 3, confidence interval 95%) is observed after >700 cycles and 2 months of testing. Extrapolating to 20% total capacity loss, this loss rate corresponds to a projected lifetime of 11,000 cycles and 2.5 years. In an effort to further improve the lifetime of the MnHCMn electrode, it was operated from –0.55 to –0.83 V, which is equivalent to a SOC range of 2.5–97.5%. After >900 1C cycles (test duration 3 months) in this range, a loss rate of 14 ± 1 p.p.m. per cycle (*N* = 4, confidence interval 95%) is observed (Supplementary Fig. 5). That improved loss rate projects to a lifetime of 14,000 cycles and 3.25 years to 80% capacity retention. We attribute the remaining capacity loss to the oxidation HCMn groups on the surface of the MnHCMn particles by a trace amount of oxygen to HCMn(III), which in turn is readily hydrolyzed^16^. Removal of the water from the cosolvent electrolyte would presumably eliminate this loss mechanism; however, the electrochemical characterization of MnHCMn(II/I) in a 1 M NaClO_4_, 100% MeCN electrolyte showed significantly poorer kinetics and lower capacity (Supplementary Fig. 6).

### Mn^1+^/Mn^2+^ redox couple in MnHCMn

The low potential cycling of our cubic MnHCMn electrode vs. SHE indicates an intriguing redox reaction in this material. Previously, the corresponding reactions at the 0 and 0.7 V potentials upon Na/Na^+^ reference electrode were assigned to carbon-coordinated Mn^III/II^(C) and nitrogen-coordinated Mn^III/II^(N), respectively^11^. The mechanism is unclear for the –0.7 V reaction that is utilized here. No data have heretofore clarified the low-potential cycling mechanism and substantiated the favorability between Mn^II/I^(C) and Mn^II/I^(N)^10,11^. The lack of understanding stems from the lack of a direct probe of the oxidation states of the Mn(C) and Mn(N) with the necessary site sensitivity.

Very recently, sXAS is demonstrated to be a site-sensitive probe of the TM redox centers in PBA materials. Such sensitivity of sXAS could clarify the N- and C-coordinated TM cation valence and spin states in PBAs^17^. sXAS probes the unoccupied electronic states in the vicinity of the Fermi level, which are closely related with the crystal fields, chemical bonds, formal valences, orbitals, and spin characters of the material^18^. sXAS is a probe with both surface and bulk sensitivity through the total electron yield (TEY) and total fluorescence yield (TFY) detection channels with about 10 and 100 nm probe depths, respectively. In particular for Mn, the TM *L*-edge sXAS directly probes the Mn-3*d* states through dipole allowed 2*p*-3*d* transitions. Such a direct probe of Mn-3*d* states lead to distinct spectral lineshapes of Mn with different oxidation states, which enables detailed analysis of the Mn valences^19–22^. Utilizing sXAS for determining Mn states in PBAs is reliable because TMs in PBAs take well-defined spin states^17^, which lead to distinct sXAS spectral lineshape^23^. In this study, we employ both sXAS and RIXS to clarify the cycling mechanism of the Mn(C) and Mn(N) groups in our cubic MnHCMn electrodes. The two sets of spectroscopic results are consistent with each other and provide the first direct experimental probe of the low-spin 3*d*^6^ state of Mn, i.e., Mn^1+^(C). In this section, we first discuss the sXAS results.

Figure 3a shows the comparison between the Mn *L*_3_-edge sXAS spectra collected on a series of cycled MnHCMn electrodes and those from multiplet calculations with site sensitivity. Atomic multiplet calculations for Mn *L*-edge sXAS are based on a previously developed model that incorporates both forward- and back-bonding between Mn and the octahedrally coordinated CN ligands^17,24^ (see methods). The calculated Mn^II^(N) *L*_3_ spectrum shows a characteristic three-peak structure with a main peak *b*. This suggests a high-spin Mn^II^ system and is consistent with other high-spin Mn^II^ systems^19^. On the contrary, the stronger crystal field of carbon-coordinated Mn(C) encourages low spin states, consistent with the previous experimental reports^25^. The lineshape of the *L*_3_ sXAS of Mn^II^(C) changes dramatically from that of Mn^II^(N) and is divided into two regions (*a* and *e* in Fig. 3a) separated by >4 eV. Especially, the very low energy of the state at 638.8 eV of Mn^II^(C) derives from the single vacant *t*_2g_ level in a low-spin 3*d*^5^ configuration. Because sXAS detects the unoccupied electron states, such a low-energy Mn^II^(C) state is to be first filled when electrons are introduced into the system (reduction). Therefore, the C-coordinated Mn^II^(C) is energetically favored to be reduced. The calculated reduced Mn^I^(C) calculation is dominated by a single *L*_3_ peak at 643.4 eV, as expected for low-spin 3*d*^6^ configurations with a fully occupied *t*_2g_ level, outside of a narrow parameter space for backhanding energetics^24^.

The experimental sXAS data display a reversible lineshape evolution upon sodium intercalation/extraction in accordance with the excellent cycling stability of the material. As discussed above, the absorption features *b* and *c* (Fig. 3a) originate from Mn^II^(N) and are of high-spin character. The high energy peaks (*d* and *e*) derive from the low-spin Mn(C). Feature *a* contains two states from the Mn^II^ contributions of both C- and N-coordinated sites, with the states of Mn^II^(C) sitting at a slightly lower energy. All the experimental spectra exhibit the predominant absorption peak *b* at 640 eV, which stems from the high-spin Mn^II^(N), suggesting that the Mn^II^(N) is not electrochemically active during the cycling around –0.7 V vs. SHE.

The most profound spectral evolution appears in the energy range of 642.5–646 eV, where the low-spin character of the Mn(C) is dominant. For all the charged electrodes (reduced, −0.9 V), the low-spin Mn(C) display a peak (feature *d*) at 643.4 eV, in contrast to the broad feature group *e* (642–646 eV) that evolves in discharged electrodes (oxidized, –0.5 V). The emerging peak *d* in the charged (reduced) electrodes aligns well with the calculated monovalent Mn^1+^ of the carbon-coordinated Mn(C), thus demonstrating that the redox reactions occur on the Mn(C) with a nominal oxidation state of Mn^II^(C) when discharged and Mn^I^(C) when charged. The overall change in the intensity of feature *a* is consistent with the cycling of Mn^II^(C)/Mn^I^(C). Because Mn^I^(C) does not contribute to the feature at about 638.8 eV, a relatively lower intensity of feature *a* is observed at the charged (reduced, Mn^I^(C)) state.

In addition to the detailed lineshape analysis of the Mn-*L*_3_ sXAS TEY data in Fig. 3a, the spectra with both the Mn *L*_3_- and *L*_2_-edges are shown in Supplementary Fig. 7. The bulk-sensitive TFY sXAS data are shown in Supplementary Fig. 8. Overall, both the full-energy range and TFY results are consistent with the detailed Mn-*L*_3_ analysis above (Fig. 3a). Although the TFY spectra are distorted by the so-called self-absorption effects, the enhanced TFY Mn^1+^(C) feature at 643.4 eV is evident in the results, suggesting that the Mn^1+^ is the intrinsic bulk state of the charged (reduced) samples.

### Direct probe of Mn^1+^/Mn^2+^ in MnHCMn

**Fig. 4:**
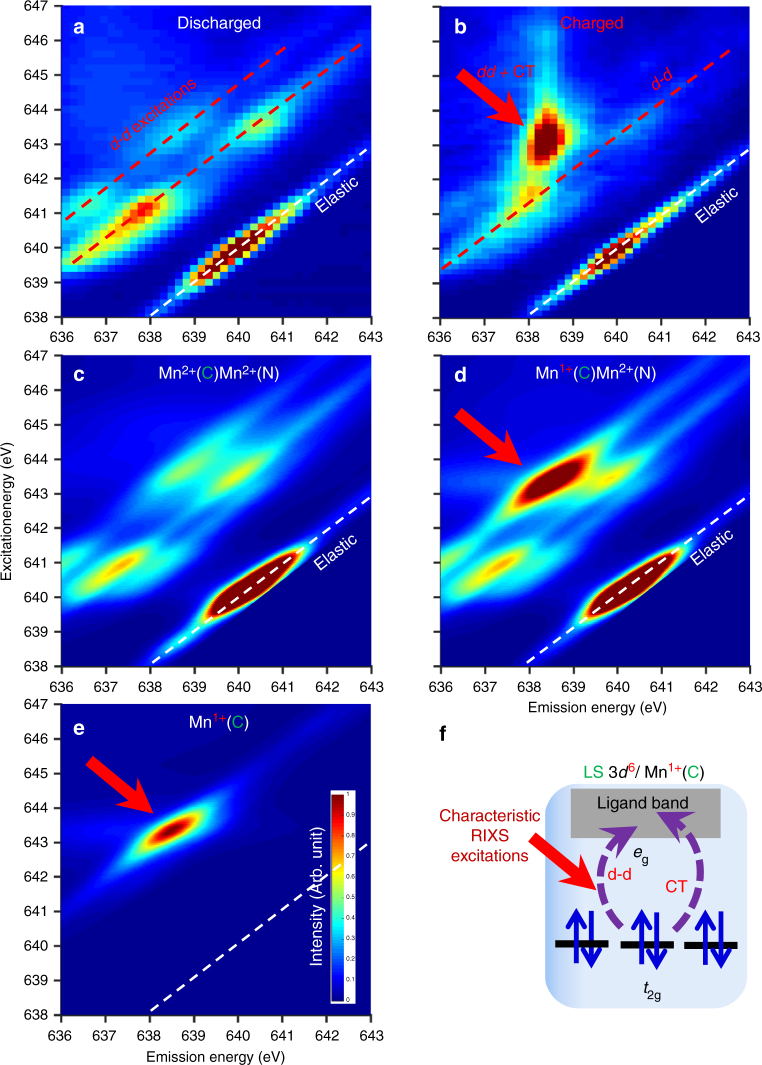
Direct probe of Mn^1+^ by soft X-ray RIXS maps. **a**, **b** Mn *L*_3_-edge RIXS maps on MnHCMn electrodes at the discharged (oxidized) and charged (reduced) states, respectively. **c**, **d** Corresponding RIXS calculations with the nominal Mn valence marked on the maps. The strong RIXS intensity of the charged electrode (red arrow) is reproduced only with Mn^1+^ at the C-coordinated site, as shown in **e**. Calculation results with other configurations are shown in Supplementary Fig. 15. **f** A schematic to explain the characteristic Mn^1+^ RIXS feature, which fingerprints the excitations from the fully occupied *t*_2g_ to the nominally vacant *e*_g_ orbitals (*d-d*) and delocalized ligand band states (CT) of a low-spin 3*d*^6^ state, i.e., Mn^1+^(C)

While the combination of the sXAS experimental and theoretical results provide strong evidence of the Mn state evolution of MnHCMn during electrochemical operation, the origin of the strong sXAS peak at 643.4 eV remains unclear and its assignment to Mn^1+^(C) relies on theoretical calculations. In order to provide direct experimental confirmation of the Mn^1+/2+^ redox couple, especially the novel Mn^1+^ state, we have employed RIXS mapping through the newly commissioned high-efficiency iRIXS system^26^. Technically, RIXS is collected by changing the incident X-ray photon energy (vertical axis in Fig. 4) across the interested absorption edge (Mn-*L*_3_ edge here) and detect the inelastically scattered photon energy (horizontal axis in Fig. 4) through a spectrometer. It has been established that RIXS features correspond to various low-energy excitations^27^. For 3*d* TM elements, RIXS signals are dominated by the strong excitations within the 3*d* states, called “*d-d* excitations” (see Chapter 8 of ref. ^28^ and many references therein). Because different numbers of 3*d* electrons (TM valence) naturally lead to different *d-d* excitations, RIXS is a sensitive probe of the novel Mn^1+^ state with further resolved emission energy that is ignored in sXAS. In a localized atomic physics model, Mn^1+^(C) is a low-spin 3*d*^6^ electron system. Because of the crystal field splitting of the five 3*d* states into *t*_2g_ and *e*_g_, Mn^1+^(C) features a fully occupied *t*_2g_ (6 electrons) and empty *e*_g_ state in particular. As schematically displayed in Fig. 4f, such a valence electron state configuration will naturally enhance the intra-3*d* excitation across the large crystal field splitting between *t*_2g_ and *e*_g_, because the full *t*_2g_ in Mn^1+^(C) does not allow other excitations within the *t*_2g_ as in Mn^2+^. This leads to an enhanced feature in RIXS with a relatively large energy loss (difference between the excitation and emission energies).

As clearly shown in Fig. 4a, b, the discharged (Mn^2+^) sample displays several groups of the *d-d* excitation features (red dashed lines in Fig. 4a) that are typical for a Mn^2+^ system with partially occupied *t*_2g_ and *e*_g_ states^29^. In sharp contrast, the charged sample displays a greatly enhanced RIXS feature with a relatively high energy-loss value (away from the elastic line for about 5 eV) at the excitation energy of about 643.4 eV. This excitation energy matches exactly the energy of the key Mn^1+^(C) sXAS feature (Fig. 3a).

This is the first time low-spin 3*d*^6^, i.e., Mn^1+^, is directly fingerprinted through such strongly enhanced RIXS excitation feature, which is expected from a localized atomic state model with fully occupied *t*_2g_ states (Fig. 4f). Furthermore, our theoretical calculation confirms such assignment by reproducing the same contrast between the Mn^2+^(C)Mn^2+^(N) and Mn^1+^(C)Mn^2+^(N) system, as shown in Fig. 4c, d, with an enhanced 5 eV energy-loss feature from the Mn^1+^(C) configuration (Fig. 4e).

However, we note that RIXS is a two-step process, and inaccuracies in the simulations of the core-hole resonance and the final low energy excitations are compounded, therefore, it is hard to get as good an agreement with experiments as for sXAS. Although our theoretical calculations include the charge-transfer (CT) channels in the form of *t*_2g_ back-bonding^17,24^, features from delocalized states are largely underestimated and the atomic picture described above considers only the localized 3*d* states. The detailed analysis of the RIXS results is not a topic of this work, but as shown in Supplementary Fig. 16, the specific Mn^1+^ feature at 643.4 eV excitation energy displays an emission energy aligned with a long broad vertical feature around 638.3 eV emission energy in the full Mn*-L* RIXS map, which is typically called fluorescence in the RIXS community and represents the coupling of local multiplets to (ligand) band-like itinerant states. This indicates that this strong Mn^1+^ feature also contains significant contributions from CT between Mn 3*d* and the ligand band states. Additionally, a cross-over from *d-d* type to non-resonant fluorescence signals is seen around 641.7 eV excitation energy, which could be due to the quantum interference effects when the excitations involve two unoccupied states close in energy, e.g., Mn from different sites. However, the intensity drop of the quasi-elastic feature around 643.4 eV excitation energy (Supplementary Fig. 16) can be interpreted by the multiplet model and is due to the absence of a low-energy spin degree of freedom in the filled *t*_2g_ shell of Mn^1+^(C). Compared with the almost purely localized *d-d* excitations in the RIXS of the discharged (Mn^2+^) system (Fig. 4a), the Mn^1+^ feature (red arrow in Fig. 4b) aligns with the fluorescence feature and displays unusually strong intensity. This suggests a strong overlap of the ligand and Mn-3*d* states, i.e., enhanced hybridization, as also elaborated in the next section.

Mn^1+^ in such coordinated compounds has been proposed since 1928^30,31^, and it was also speculated in other type (monoclinic/rhombohedral) of MnHCMn electrodes^10,11^; our consistent results of sXAS and RIXS here finally provide the long-awaiting experimental verification.

### Strong hybridization and covalency in MnHCMn

A quantitative simulation of the sXAS of the fully charged and discharged MnHCMn samples is provided in Supplementary Fig. 9 through a linear combination of the calculated Mn^II^(N), Mn^II^(C), and Mn^I^(C) spectra. The lineshape evolution upon the SOC can be seen from the simulated spectra that are in good agreement with the experimental results, which testifies the validity of our theoretical calculations. Interestingly, the calculated *d* electron occupancies, as listed in Supplementary Table 1, for Mn^II^(C) and Mn^I^(C) ions are 4.658 and 5.530, respectively. These values are much lower than the nominal values of 5 and 6 for Mn^2+^ and Mn^1+^. Such divergences of the electron occupation numbers indicates a strong covalency in the system, in which the hybridization between the Mn 3*d* and the C-N molecular orbitals results in the delocalization of the Mn*-*3*d* valence electrons^32^.

Because the electron delocalization is a critical parameter to determine the electronic conductivity, which is directly related with the rate performance of a battery material, we further testify the scenario of the strong hybridization and delocalization in the MnHCMn system by measuring the N-2*p* electron states through the N-*K* sXAS. Figure 3b displays the bulk-sensitive TFY signal of N*-K* sXAS of the same batch of cycled electrodes. The predominant absorption feature is located around 400 eV and can be assigned to transitions from N 1*s* core electrons to the unoccupied CN π* orbitals^32,33^. This feature is shifted slightly to higher energy for the discharged (oxidized) electrodes as compared with those of the charged (reduced) ones, consistent with the overall chemical potential change in the system. The most profound spectral evolution is found at around 397.5 eV, where the discharged (oxidized) electrodes exhibit an absorption peak that is attributed to the hybridized state of CN π* with the Mn 3*d* orbitals^32^. The variation of this low-energy N*-K* feature upon SOC is also shown in the surface-sensitive TEY spectra (Supplementary Fig. 10), but the stronger effect in the TFY data (Fig. 3b) indicates that this is a bulk property of the discharged (oxidized) MnHCMn.

Although a similar evolution in the C *K*-edge sXAS spectra are expected, the C *K*-edge data (Supplementary Fig. 11 and 12) are dominated by the signals from carbon black and binder on the surface of the composite electrodes (Supplementary Fig. 13). Nonetheless, the bulk-sensitive TFY data (Supplementary Fig. 11) show a weak but clear low-energy state only in the discharged (oxidized) samples, resembling that of the N-*K* lineshape evolution. During the Na^+^-intercalation charging (reducing) process, these low-energy hybridized state is favored in energy and gets filled in conjunction with the Mn 3*d* orbital, as indicated by its disappearance in the N*-K* and C*-K* sXAS data of the charged electrodes (Fig. 3b, S11).

The evolving low-energy features in the N*-K* and C*-K* sXAS data reveal the strong hybridization and thus the delocalization of the Mn-3*d* electrons. Additionally, the Mn^1+^ RIXS features also indicate strong overlap of the ligand and Mn-3*d* states, as briefly described above and will be detailed elsewhere. These evidences are consistent with the much lower Mn-3*d* orbital occupancy compared with the nominal values (Supplementary Table 1). The strong hybridization of the CN molecular and Mn 3*d* orbitals implies that the electron states in the MnHCMn material are intrinsically delocalized, which will fundamentally enhance the electron transport and improve the performance of the electrode at high cycling rates.

### Co-solvent electrolyte and PBA solubility

**Fig. 5:**
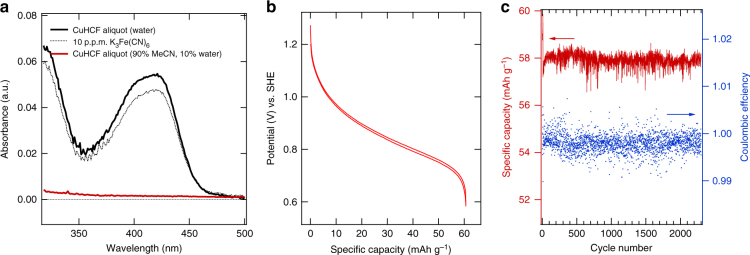
Dissolution and half-cell tests of CuHCF in co-solvent electrolyte. **a** The UV-vis spectra of aliquots of water and the 90% MeCN + 10% water cosolvent electrolyte, in which a CuHCF electrode was stored at room temperature for at least 48 h. A 10 p.p.m. K_3_Fe(CN)_6_ standard sample is measured for comparison. The peak at 420 nm corresponds to the dissolved Fe(CN)_6_^3–^. No dissolution is observed for the co-solvent electrolyte. **b** The reaction potential profile of the CuHCF cathode at a 1C rate with the co-solvent electrolyte. **c** The cycle life and coulombic efficiency of the CuHCF electrode during 1C–1C cycling between 0.6 and 1.15 V over 6 months. The noise is due to the precipitation of salt in the Ag/AgCl reference electrode frit, resulting in an inconsistent junction potential between the reference electrode-filling solution and the electrolyte

While PBA-based electrodes are stable and of low cost, the material dissolution into aqueous electrolyte severely limits the lifetime of PBA electrodes, as reported previously for CuHCF^6,13^. Previously, electrolyte salt concentration was tuned to mitigate the solubility issue of CuHCF^7^. Here an aqueous–organic cosolvent electrolyte was developed to solve this problem. We employ ultraviolet-visual spectroscopy (UV-vis) to characterize the dissolved ferricyanide in aqueous and organic–aqueous electrolytes. Figure 5a shows the clear contrast on the solubility of CuHCF between the water and the 90% MeCN:10% water electrolyte. After the CuHCF electrode is soaked in the electrolyte for >48 h, the charged CuHCF has a solubility of >10 p.p.m. in water; however, in the organic–aqueous co-solvent solution, zero dissolution is detected. Electrochemical characterization of CuHCF cathode with our cosolvent electrolyte is performed in half-cells containing a 1 M NaClO_4_ cosolvent electrolyte (90% MeCN, 10% water). Figure 5b displays a solid–solution reaction centered at 0.85 V vs. SHE and providing 62 mAh g^−1^ was observed, which is consistent with the oxidation and reduction of the hexacyanoferrate groups as previously observed in aqueous electrolytes^6^. The coulombic efficiency of the cycling is 99.9%. During cycling at a symmetric 1C rate (CCCV charge, CV step to C/10) between 0.6 and 1.15 V, which corresponds to a SOC range of approximately 0–95%, a specific capacity of 58 mAh g^–1^ was observed. Extended half-cell cycling under these conditions resulted in >2300 cycles with zero capacity loss during over 6 months of testing (Fig. 5c). This is in sharp contrast with the CuHCF electrode cycled in aqueous electrolyte, which typically shows clear capacity decrease within several days (Supplementary Fig. 17). This superior long cycle- and calendar-life was achieved because of the insolubility of CuHCF in our co-solvent electrolyte, which stands in contrast to the relatively short calendar lives of PBA cathodes in aqueous electrolytes^6,13^.

**Fig. 6:**
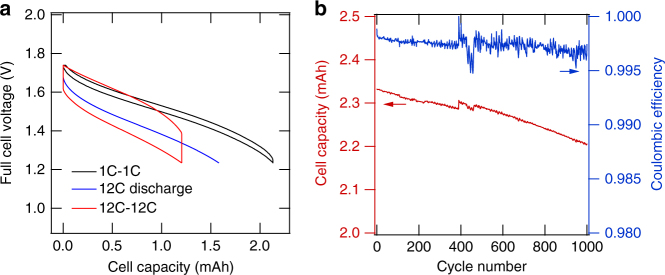
Na-ion battery full-cell tests based on CuHCF cathode, MnHCMn anode, and co-solvent electrolyte. **a** The voltage profiles of the full CuHCF/MnHCMn cell during 1C–1C cycling, 12C discharge from 1.74 V, and 12C-12C cycling. The full-cell-specific energy and energy density are 33 Wh kg^–1^ and 66 Wh L^–1^, respectively. **b** The cycle life and coulombic efficiency of the full cell during 1C–1C cycling between 1.74 and 1.23 V. For the first 400 cycles, galvanostatic charging with no potential hold (CV step) was used. After cycle 400, a CV step to a C/10 current, typically of 1–2 min in duration, was performed. The oscillations in capacity and coulombic efficiency are diurnal and correspond to temperature fluctuations from 23 to 25 °C in the cell testing station

*An all-PBA low-cost Na-ion battery full cell*. Full pouch cells containing the MnHCMn anode, the CuHCF cathode (1.5× capacity), and the cosolvent electrolyte were cycled between 1.74 and 1.23 V (Fig. 6a). This full-cell voltage range resulted in 95% anode capacity utilization, 99.8% coulombic efficiency, and 97.7% round-trip energy efficiency during symmetric 1C cycling. During a 12C discharge from full charge, the cell delivers 74% of its total energy >1.23 V. Even with the symmetric 12C cycling, the cell still provides 55% of its total energy with 88% round-trip energy efficiency. The lost energy is due to the high internal resistance of the prototype pouch cell, which contained a mesh reference electrode between the active electrodes. We expect that the optimization of cell design for low impedance will further improve the energy retention during rapid cycling. At the 1C rate, by assuming 100% capacity utilization, 2/3 active cell mass, and 2 g mL^–1^ cell density, the full cell delivers a theoretical specific energy and energy density of 33 Wh kg^–1^ and 66 Wh L^–1^, respectively. We note that engineering optimization of the battery device is necessary to achieve this goal. Although this energy density is lower than that of Li-ion cells, it is comparable to that of the lead acid cells presently used in stationary storage systems.

After 1000 cycles at 1C (3.5-month test duration) utilizing 95% of the total anode capacity, the full cell retains 95% of its initial discharge capacity, which projects to 4000 cycles to 80% capacity retention (Fig. 6b). The full-cell capacity loss rate of 50 p.p.m. per cycle is approximately three times the capacity observed during the half-cell anode test. The higher loss rate observed in the full cell is attributed to at least two sources. First, oxygen may slowly diffuse into even a well-sealed pouch cell due to the permeability of the polymer laminate to oxygen. Additionally, it is possible that trace amount of oxygen is generated at the cathode at high potentials and migrates to the anode. Both effects could result in the oxidation of the active material surface to HCMn(III), which may react with water to form manganese oxide phases^16^. Second, any imbalance in the coulombic efficiencies of the two electrodes results in a “slip” in their relative SOC ranges, which results in a decrease in the accessible full-cell capacity within the fixed upper and lower voltage cutoffs. Nonetheless, the full cell data shown here represent a full Na-ion battery cell that achieves high-rate and long-life electrochemical cycling and with space for further optimizations in design and balancing.

Furthermore, a recent analysis of Li-ion cells showed that the active materials comprised about 50% of the total manufactured cell cost^34^. For the all-PBA cells here, the material cost is significantly lower. The other manufacturing costs will be further lowered because the electrolyte contains water, so the costly dry room is not required (see P.A. Nelson et al.^35^, Modeling the Performance and Cost of Lithium-Ion Batteries for Electric-Drive Vehicles, 2011). Therefore, the developed all-PBA Na-ion battery system provides a unique opportunity for dramatically decreasing the cost for grid-scale energy storage (see Supplementary Notes 1 and 2 for detailed cost estimation).

## Discussion

We report a Na-ion battery full-cell system based on a novel MnHCMn(II/I) anode, CuHCF cathode, and an organic–aqueous cosolvent Na^+^ electrolyte. The full cell displays excellent energy efficiency and rate capability, as well as very long cycle life, at a low cost. The organic–aqueous cosolvent electrolyte solves the long-standing problem of PBA material dissolution and significantly extends the practical calendar lifetime of the electrodes. The MnHCMn(II/I) anode operates through a novel mechanism, in which carbon-coordinated manganese is reduced to a monovalent Mn^1+^ with a low-spin 3*d*^6^ oxidation state at full charge, as verified for the first time using sXAS and RIXS. The all-PBA Na-ion system inherently benefits from the low cost, structural stability, and high kinetics of the materials and provides a unique solution for stable high-rate low-cost grid-scale energy storage.

In a typical chemical environment, divalent Mn is reduced directly to Mn^0^ with no intermediate monovalent state^36^. Hexacyanomanganate is one of the coordination complexes in which the Mn^1+^ has been expected from chemical analysis and electrochemical reduction for nearly a century^30,31^ but remains elusive due to the lack of experimental verifications. Our sXAS and RIXS results clarifies the existence of Mn^1+^ in the PBA-based coordination complex. The novel Mn^1+^/Mn^2+^ redox couple enables MnHCMn to be an excellent Na-ion battery anode. Furthermore, we reveal the electron delocalization through a strong Mn-3*d* and CN ligand hybridization, which indicates that MnHCMn is fundamentally favored to be an electrode material for high-rate performance. The demonstration of the Mn^1+^/Mn^2+^ redox reaction in the MnHCMn anode also inspires explorations of other novel oxidation states in coordination compounds for battery technology.

## Methods

### Materials synthesis

The synthesis of CuHCF was performed by the addition of 720 mL of aqueous 0.87 M K_3_Fe(CN)_6_ to 1080 mL of aqueous 1.00 M CuSO_4_·5H_2_O under constant stirring. All chemical precursors were Sigma Aldrich reagent grade (96–99%). After further stirring for 3 h, the material was filtered, washed with water, washed with methanol, and dried under vacuum at 80 °C overnight, resulting in 255 g brown solid product.

MnHCMn was synthesized following the same procedure as that for CuHCF. During constant stirring under a N_2_ atmosphere, 515 mL of aqueous 3.6 M NaCN was added to 600 mL of aqueous 1.2 M Mn(OAc)_2_·4H_2_O. After additional stirring for 3 h, the precipitate was filtered, washed with water, washed with methanol, and dried under vacuum at 60 °C overnight resulting in 103 g blue solid product.

### Electrochemical tests

Electrodes were prepared by mixing 80% (wt.) active material, 10% carbon black (Timcal C65), and 10% polyvinylidene difluoride binder (Kynar HSV-900) in 1-methyl-2-pyrrolidinone using a planetary centrifugal mixer. Slurries were coated on carbon-cloth substrates (Avcarb 1071HCB) and dried under vacuum at 80 °C for 2 h. Typical electrode mass loadings were 10–20 mg cm^–2^ (0.6–1.2 mAh cm^–2^). Electrode areas were 4–8 cm^2^.

Half-cell testing was performed in flooded three-electrode full cells containing a PBA cathode or anode working electrode, a PBA anode or cathode counter electrode having 3× excess capacity, excess 1 M NaClO_4_, 90% (volume) acetonitrile, 10% water electrolyte, and a Ag/AgCl reference electrode (4 M NaCl, Pine Instruments). Full-cell testing was performed in vacuum-sealed pouch cells containing a cellulosic separator and a half-charged Prussian blue reference electrode that operated as an observer. The cathode mass was about 40 mg and the anode mass was about 60 mg. The cell with a voltage range of 1.9–1.1 V allows the cathode to deliver 100% of its specific capacity (60 mAh g^–1^). Cells were vacuum-sealed under N_2_. The CuHCF cathode was pre-reduced to a SOC matching that of the anode using a cell containing the same electrolyte as above and an activated charcoal counter electrode. Cell testing was performed using a Bio-Logic potentiostat and Arbin Instruments battery testers.

### SEM and UV-vis methods

SEM was performed using a Zeiss Gemini Ultra-55 field emission microscope (5 kV beam tension). UV-vis was performed using a Shimadzu UV-3600 spectrophotometer (10 mm cuvette). To prepare UV-vis samples, a 1 cm^2^ electrode containing approximately 10 mg active material was stored in 10 mL of solution at room temperature for 48 h, and the supernatant was transferred to the UV-vis cuvette after centrifugation.

### Soft X-ray spectroscopy methods

sXAS was performed at Beamline 8.0.1 of the Advanced Light Source (ALS) in Lawrence Berkeley National Laboratory. The experimental energy resolution is about 0.1–0.15 eV. The data were collected in both TEY and TFY mode with the probing depth of about 10 and 100 nm, respectively. All the spectra have been normalized to the beam flux measured by a gold mesh. Electrode samples prepared for sXAS analysis contained 90% MnHCMn, 5% carbon, and 5% binder. They were cycled at 1C (CCCV charge to C/10) between –0.5 and –0.9 V vs. SHE in the same electrolyte as above. After cycling, they were washed with pure acetonitrile and dried under N_2_.

RIXS was performed in the newly commissioned iRIXS system at the BL8.0.1 of ALS^26^. High-efficiency spectrograph was utilized for the RIXS mapping^37^. RIXS spectra were taken with 0.2 eV step in excitation energies, the excitation energy value was first calibrated, then the emission energy was calibrated based on the elastic feature.

To avoid oxidation during sample handling, a gas-tight sample transfer chamber was used to transport samples from a glove box to the sXAS vacuum chamber without exposure to air. Samples are carefully checked for radiation damage effect. An oxidation of the low-valence Mn state (sodiated/charged samples) is observed under soft X-rays, but the high-valence Mn samples are relatively stable (Supplementary Fig. 14). Samples are itinerant during data collection with the speed of 0.2 mm s^–1^ with the beam size of about 30 µm along the motion direction. Liquid-N_2_ cooling, X-ray beam flux, and exposure time have been controlled to avoid irradiation effect in the reported data, as indicated by the contrast of the data themselves.

### Theoretical calculations

Atomic multiplet calculations for sXAS incorporates both forward- and back-bonding between Mn and the octahedrally coordinated CN ligands based on a previously established model^17,24^. The configuration-averaged energy of valence states without a core hole is set by parameters EG2 = *E*_0_−*E*_L−_ and EG3 = *E*_L–_−*E*_L+_. The terms *E*_0_, *E*_L__−_, and *E*_L+_ are the configuration averaged energies with nominal e^–^ number, with a single e^–^ transferred from the metal site to the from the surrounding cyano groups, or with a single hole transferred from the surrounding cyano groups, respectively. Identically defined parameters EF2 and EF3 apply to the core hole states.

The RIXS scattering process was modeled using the Kramers–Heisenberg equation, with inverse lifetimes of *Γ* = 1.1 eV for Mn^I^, and *Γ* = 0.8 eV for Mn^II^. The matrix elements of both RIXS and XAS were obtained through full diagonalization of the Hamiltonian, which was performed using LAPACK drivers^38^. Gaussian resolution broadening with a full width at half maximum (FWHM) of 0.5 eV was applied along the energy loss axis, and Lorentzian broadening with a 2 eV FWHM was applied at energy loss values above 5.5 eV, to represent the rapid delocalization of high-energy atomic multiplet states due to itinerancy.

The configuration energies used to describe Mn^II^(C) are EG2 = 3.26 eV, EF2 = 2.76 eV, EG3 = −3.20 eV, and EF3 = −1.2 eV. Mixing parameters are 1.2 eV for σ back-bonding, 1.92 eV for π back-bonding, 2.28 eV for σ bonding, and 0.0 eV for π bonding. The crystal field strength was set to 10 Dq = 3.9 eV. These parameters are nearly identical to the optimized parameter set for Fe(CN)_6_^4−^^17^.

Configuration energies for Mn^I^(C) are EG2 = 4.76 eV, EF2 = 3.26 eV, EG3 = −6.20 eV, and EF3 = −4.2 eV. These values represent a 1.5 eV increase in electron affinity compared to the Mn^II^(C), and a core hole monopole potential that is weaker by 1 eV relative to *d-d* Coulomb repulsion. Mixing parameters were kept identical for both calculations, and the crystal field 10Dq value was set to 4.6 eV for Mn^I^(C) to account for electron cloud expansion in a higher 3*d* occupancy.

Configuration energies for Mn^II^(N) are EG2 = –0.06 eV, EF2 = –0.44 eV, EG3 = 2.0 eV, and EF3 = 0 eV. Mixing parameters are 1.1 eV for σ bonding and 0 eV for π bonding and back-bonding. The octahedral crystal field was set to 10 Dq = 0.5 eV.

### Data availability

The data that support the findings of this study are available from the corresponding authors upon reasonable request.

## Electronic supplementary material

Supplementary Information
