# Supplementary material for: Monovalent manganese based anodes and co-solvent electrolyte for stable low-cost high-rate sodium-ion batteries
Source: Nat Commun. 2018 Feb 28;9:861. doi: 10.1038/s41467-018-03257-1 (PMC5830409; doi:10.1038/s41467-018-03257-1)
Supplement: Supplementary file 1 — Supplementary Information [file 41467_2018_3257_MOESM1_ESM.pdf]

Supplementary Figures

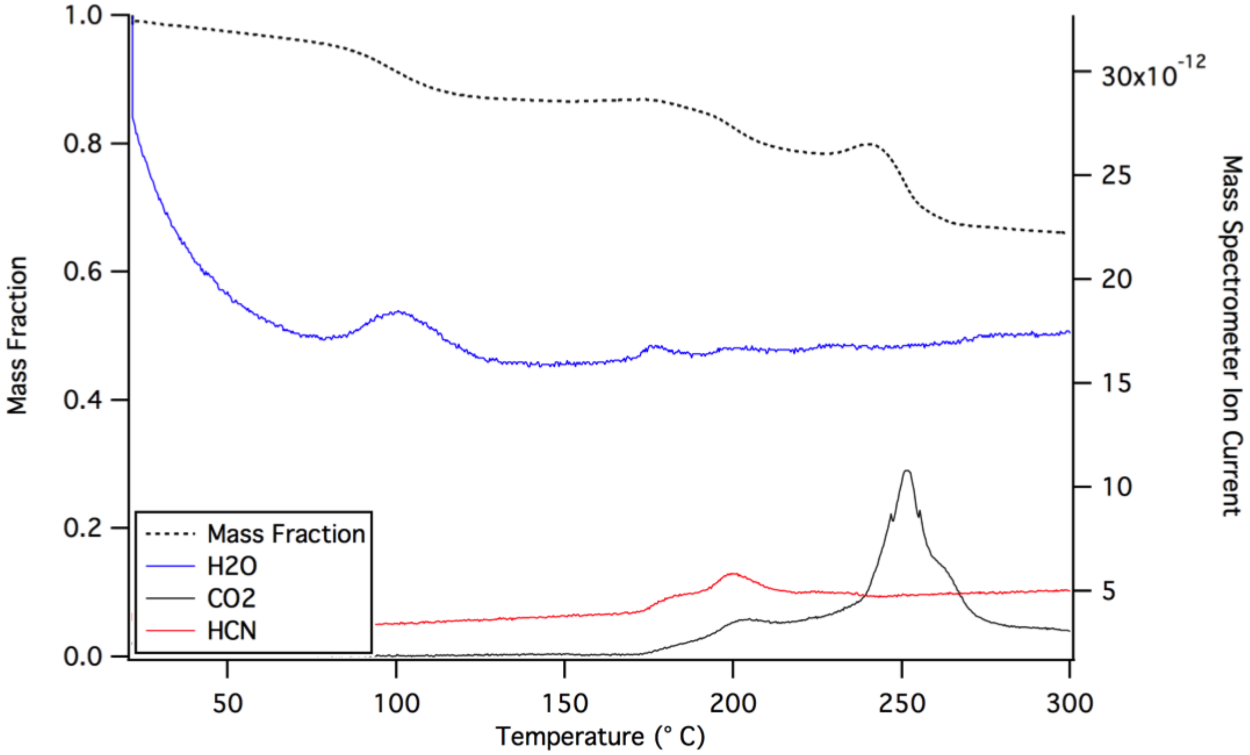

Supplementary Figure 1 The TGA-MS spectrum of the as-synthesized MnHCMn anode powder heated at 3 K min^{-1} in dry air. Due to rapid oxidation to MnHCMn(III), HCN decomposition begins at 175° C. Water loss below 150° C results in 13% mass loss.

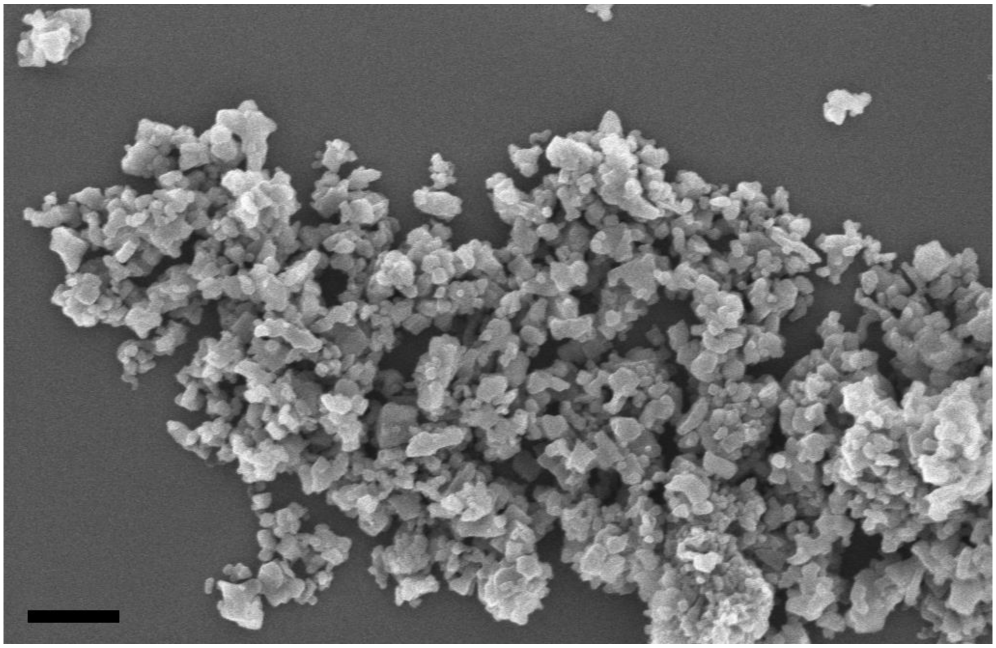

Supplementary Figure 2 SEM of the CuHCF cathode. Scale bar: 500 nm.

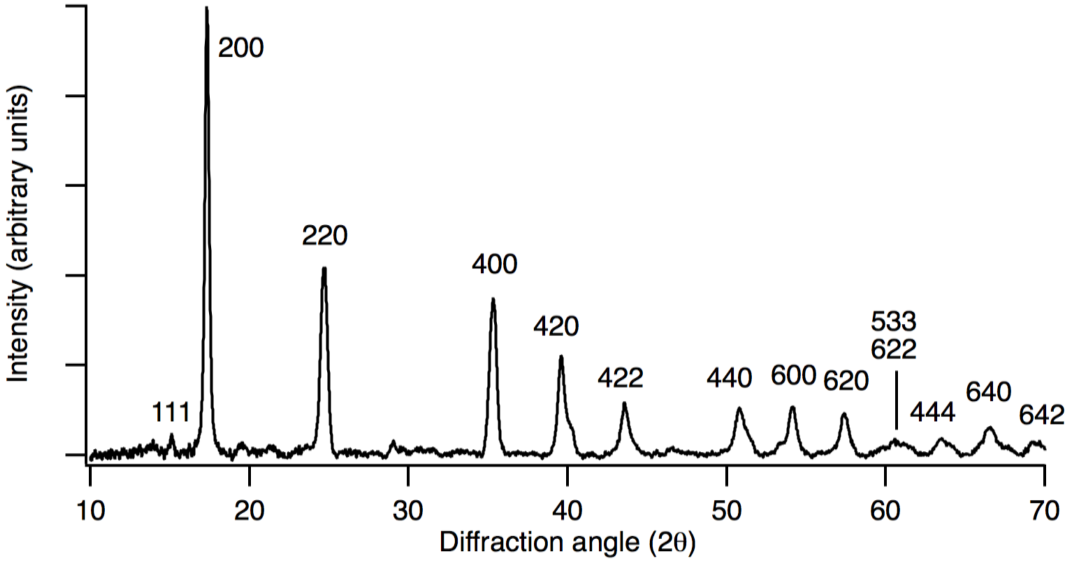

Supplementary Figure 3 The powder X-ray diffraction spectrum of CuHCF.

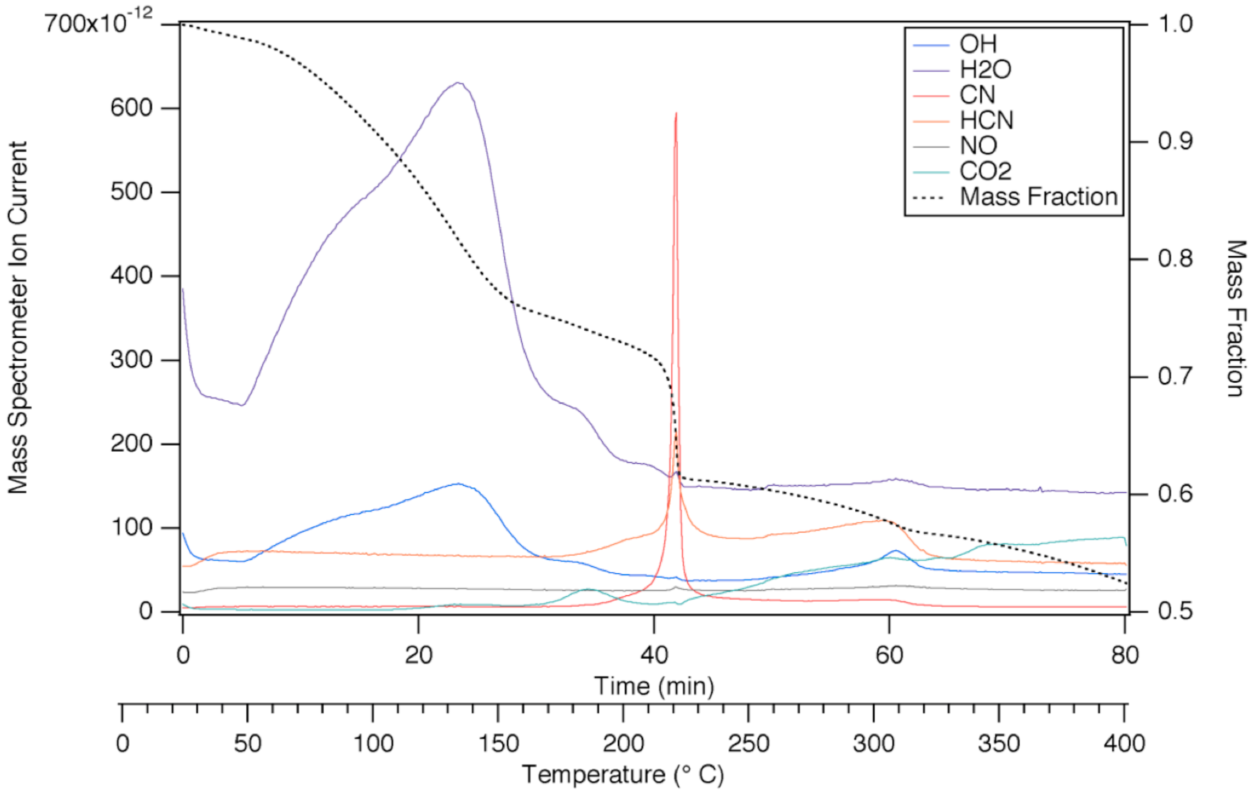

Supplementary Figure 4 The TGA-MS spectra of CuHCF heated at 5 K/minute in dry air. Water loss below 150° C results in 25% mass loss. HCN release onsets sharply at 220° C.

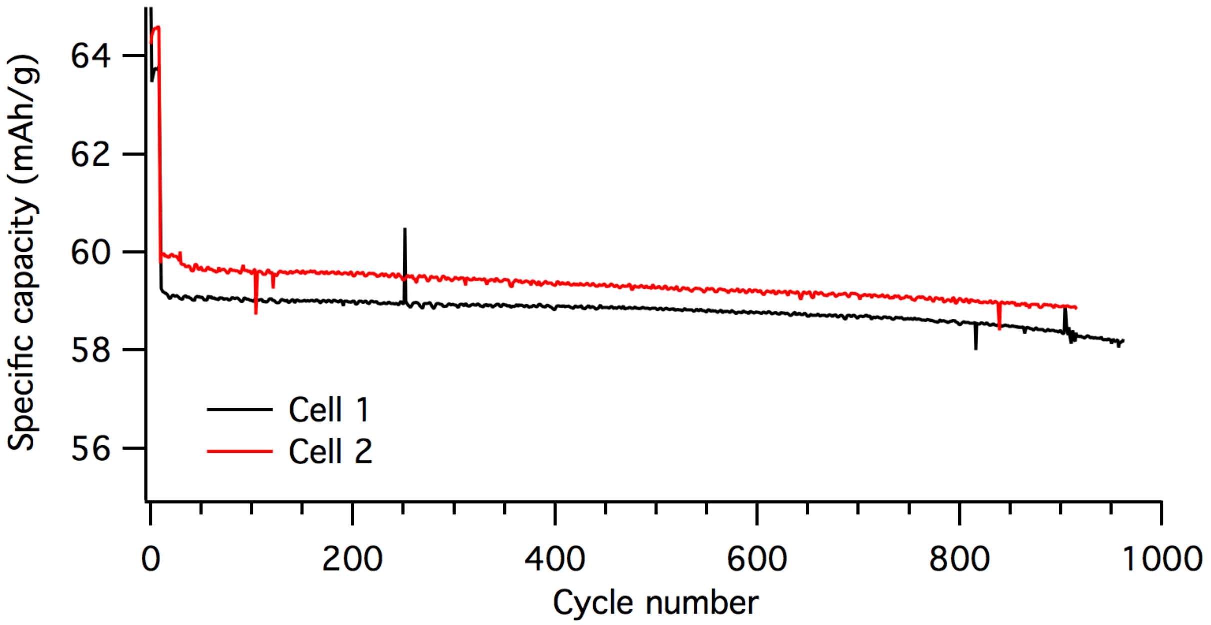

Supplementary Figure 5 The cycle life of the MnHCMn anode during 1C-1C cycling between –0.55 and –0.83 V (2.5% to 97.5% SOC). An approximately linear loss at a rate of 14 ppm per cycle is observed during 900 cycles (3 months) of testing. Both cells are tested under the same conditions, including the potential range.

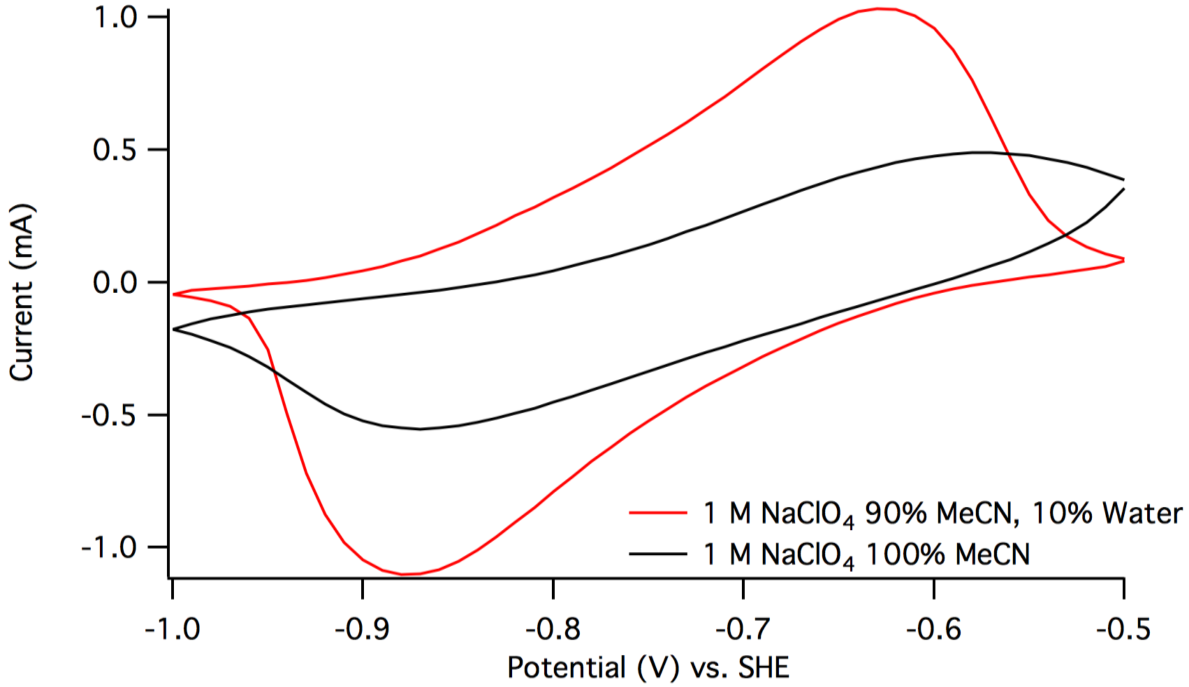

Supplementary Figure 6 Cyclic voltammograms of the MnHCMn(II/I) anode in 1 M $NaClO_4$ electrolytes. Lower signal response and slower kinetics are observed in the pure MeCN electrolyte (black), as compared to the 90% MeCN, 10% water electrolyte (red). All potential sweep rates are 1 mV s^{-1}. Electrode masses are approximately 10 mg.

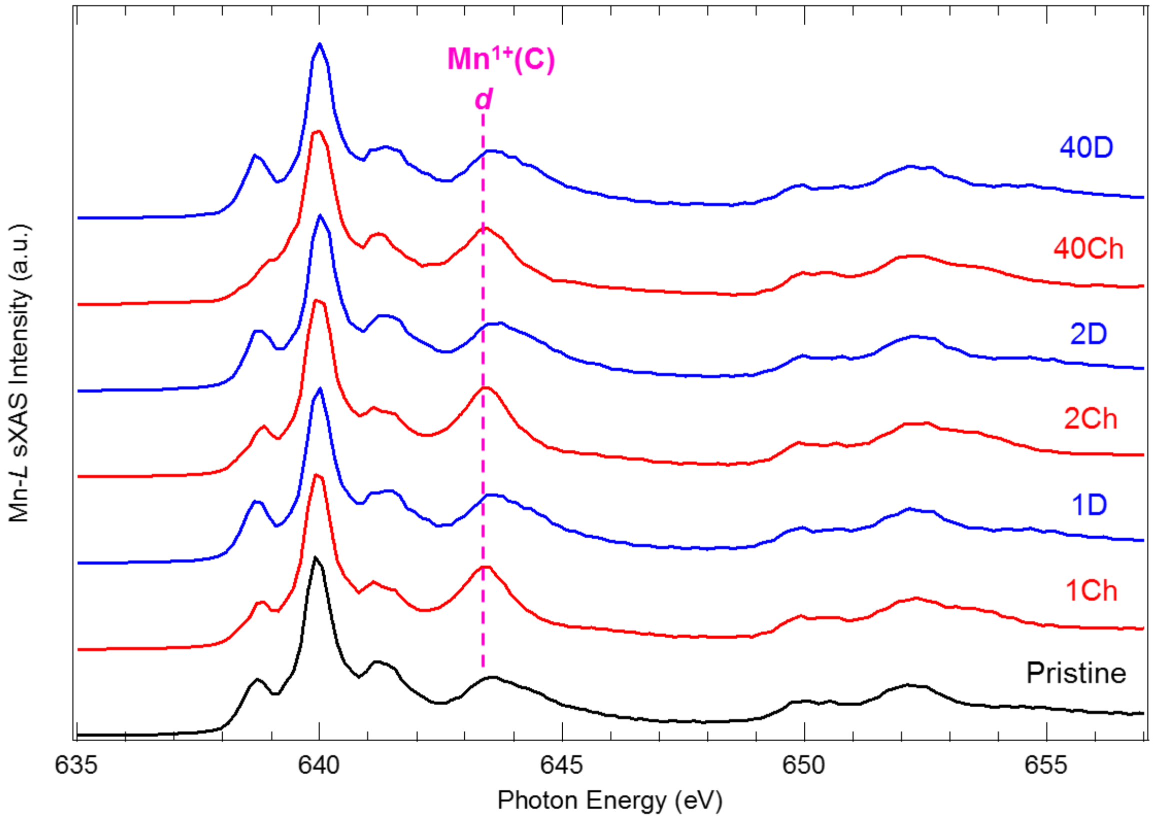

Supplementary Figure 7 Full energy range Mn $L_{3,2}$-edge sXAS spectra (TEY) collected on a series of MnHCMn electrodes (pristine, fully charged, or fully discharged after 1, 2, or 40 cycles). The L_2-edge display much broader absorption feature than L_3-edge due to the shorter lifetime of the $2p_{1/2}$ core hole as a consequence of Coster-Kronig Auger decay[1].

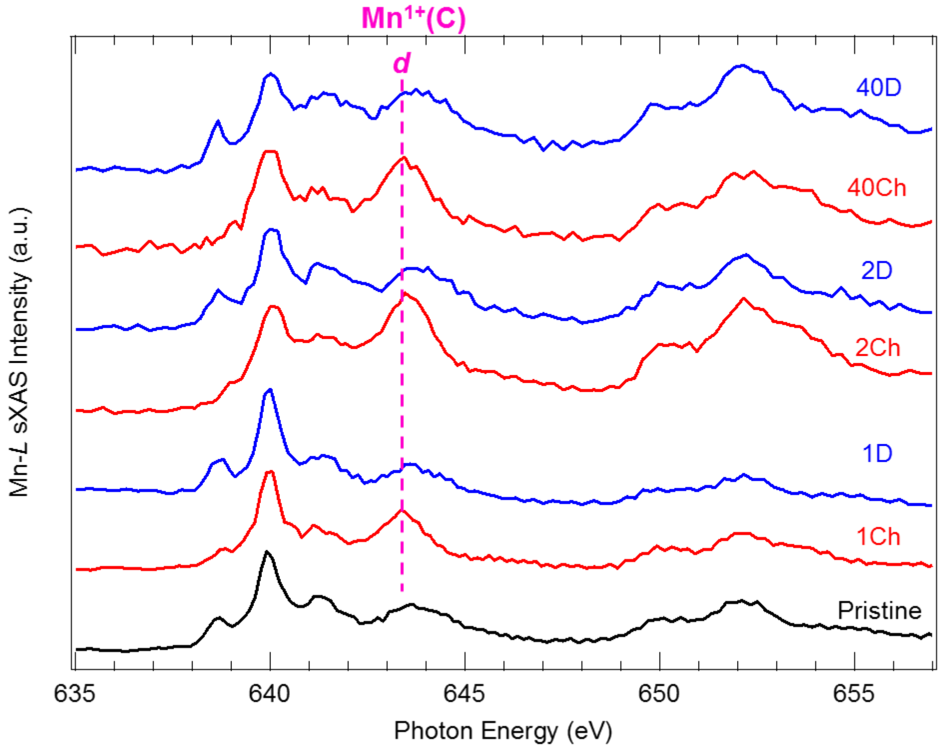

Supplementary Figure 8 Mn *L*-edge sXAS spectra (TFY with probe depth of about 100 nm) collected on a series of MnHCMn electrodes (pristine, fully charged, or fully discharged after 1, 2, or 40 cycles).

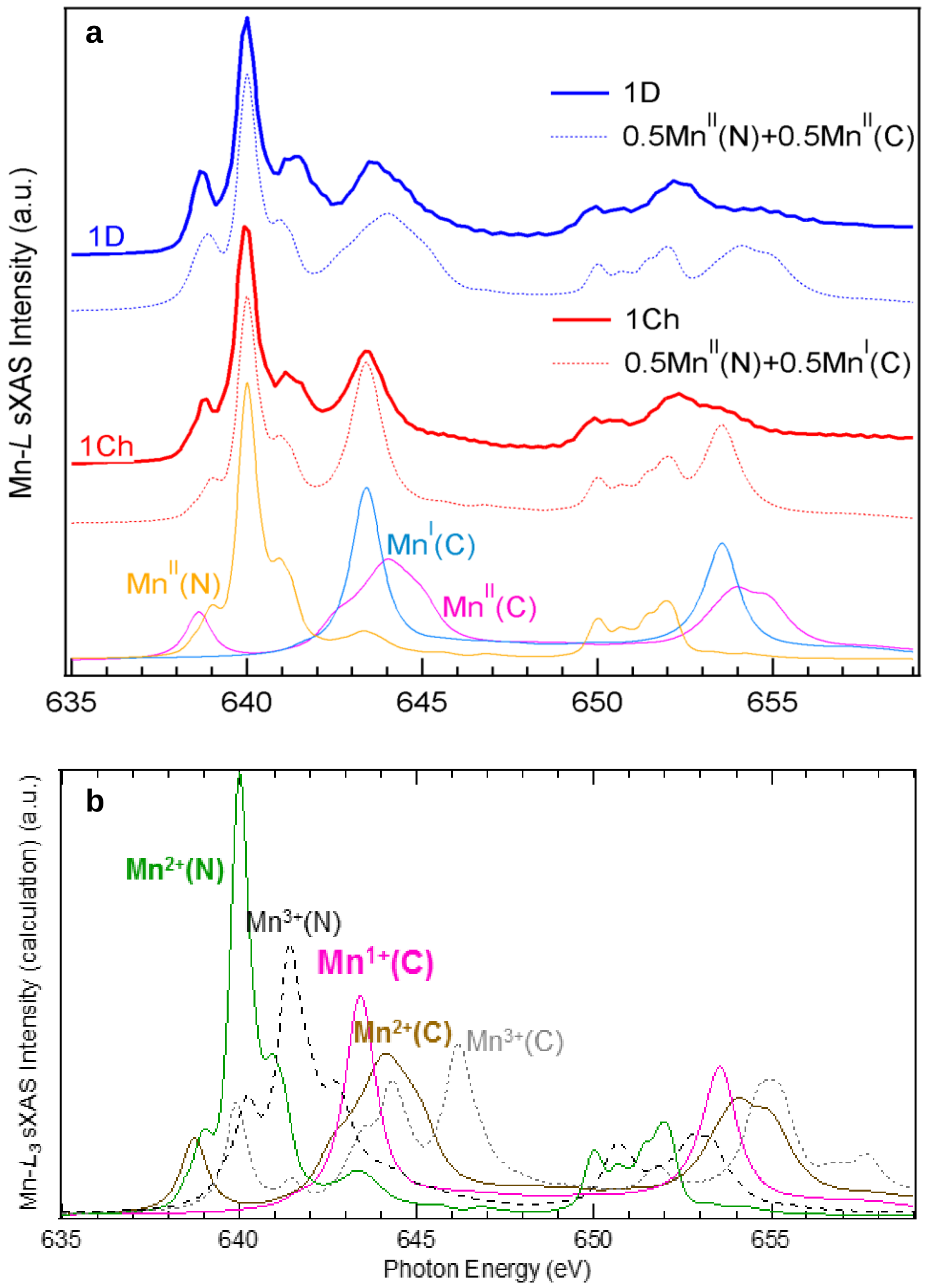

Supplementary Figure 9 (a) Experimental Mn *L*-edge sXAS TEY spectra of the MnHCMn electrodes at 0% (1D) and 100% (1C) state of charge (thick solid), and the fitted spectra (dotted). The fitted spectra are from a linear combination of the calculated Mn^{II}(N), Mn^{II}(C), and Mn^{I}(C) spectra that are shown at the bottom of the figure. (b) Calculated spectra of all possible Mn oxidation states with specific coordinations.

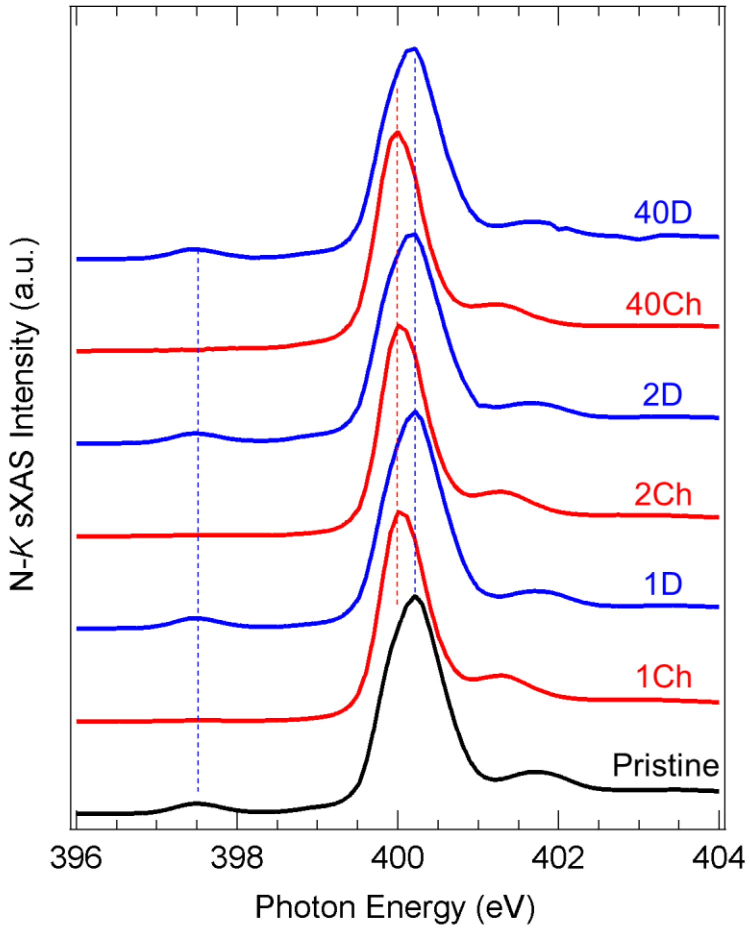

Supplementary Figure 10 N *K*-edge sXAS spectra (TEY) collected on a series of a pristine MnHCMn electrode and electrodes cycled to 0% (D) or 100% (Ch) charge (1, 2, or 40 cycles). Compared with the bulk probe of sXAS through the TFY channel (Fig. 3b), the low-energy unoccupied state at 397.5 eV in the discharged samples are much weaker, indicating that this electronic state is the material bulk property.

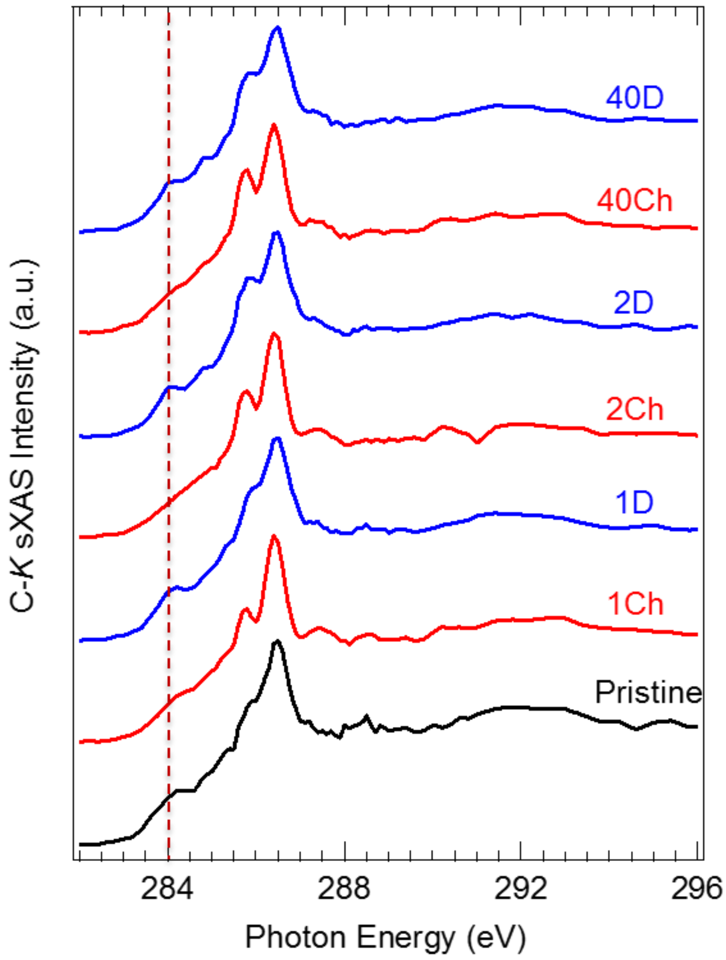

Supplementary Figure 11 C *K*-edge sXAS spectra (TFY) collected on a series of a pristine MnHCMn electrode and electrodes cycled to 0% (D) or 100% (Ch) charge (1, 2, or 40 cycles). Red line indicates the key evolution of a low-energy C-*K* feature that emerges in discharged (oxidized) samples.

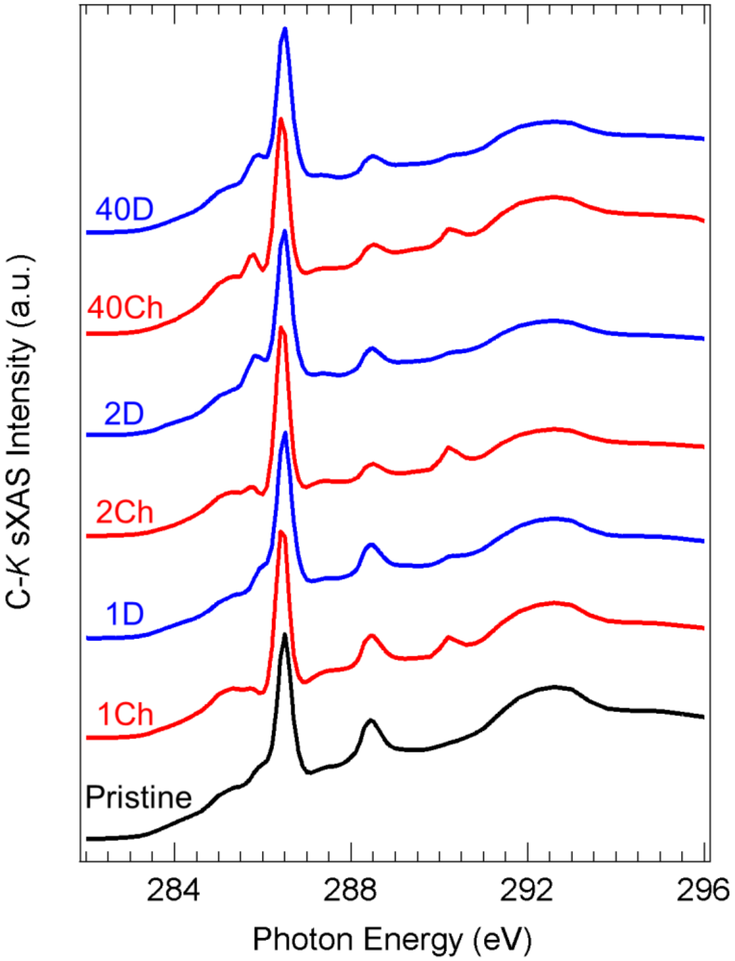

Supplementary Figure 12 C *K*-edge sXAS spectra (TEY) collected on a series of a pristine MnHCMn electrode and electrodes cycled to 0% (D) or 100% (Ch) charge (1, 2, or 40 cycles). No obvious evolution is observed due to the excessive amount of other Carbon species that coat the electrode material, e.g., binder, conductive carbon, etc. (Supplementary Figure 13).

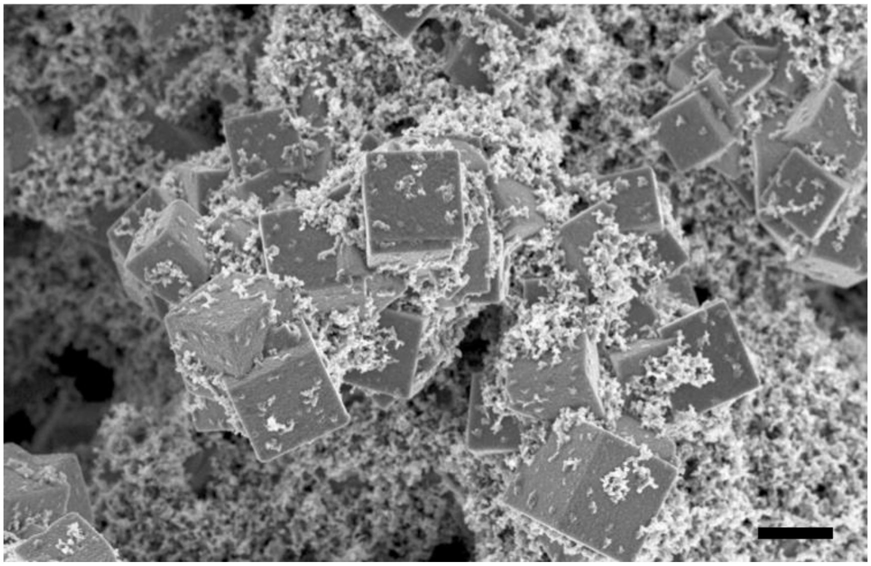

Supplementary Figure 13 SEM of a pristine MnHCMn anode. Scale bar: 2 µm. The anode particles are bound surrounded by nanocarbon black, resulting in good electronic contact and rapid cycling kinetics. The presence of the carbon black results in a large background signal in the sXAS C *K*-edge spectra, preventing analysis of the effect of charge state on the carbon in the MnHCMn structure.

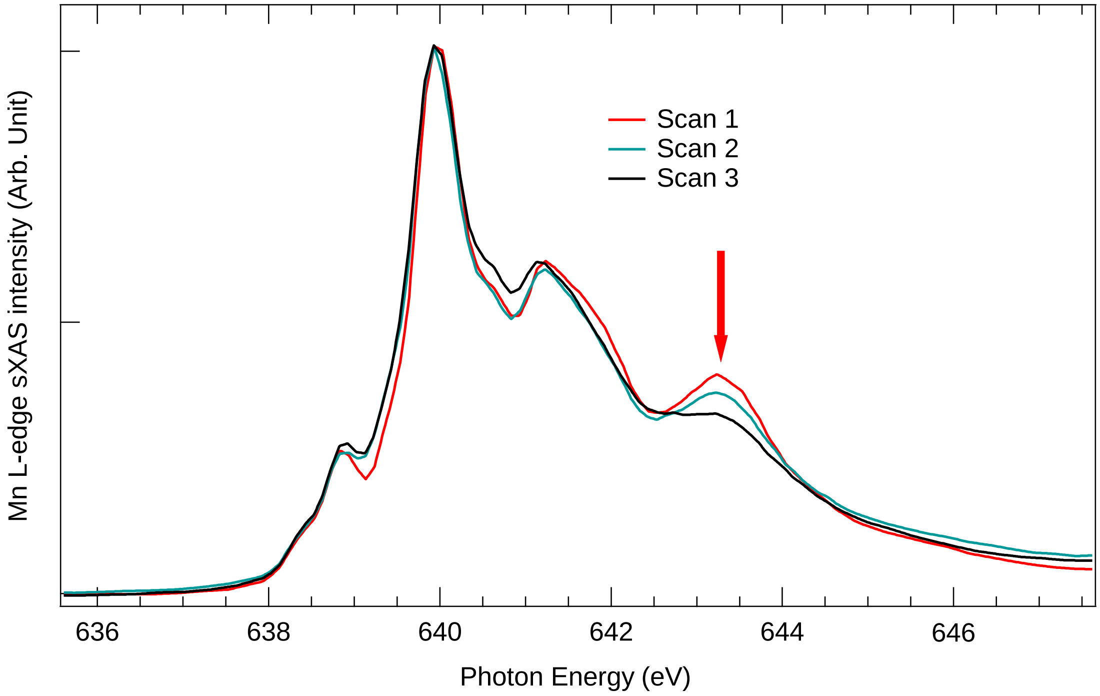

Supplementary Figure 14 Soft X-ray Radiation damage tests of charged MnHCMn anode. Three XAS scans were collected with high-flux beam hitting exactly the same spot on the sample. The intensity of the fingerprinting feature of Mn^{1+} at 643.4 eV decreases upon irradiation, indicating the Mn^{1+} could be oxidized under strong soft X-ray beam.

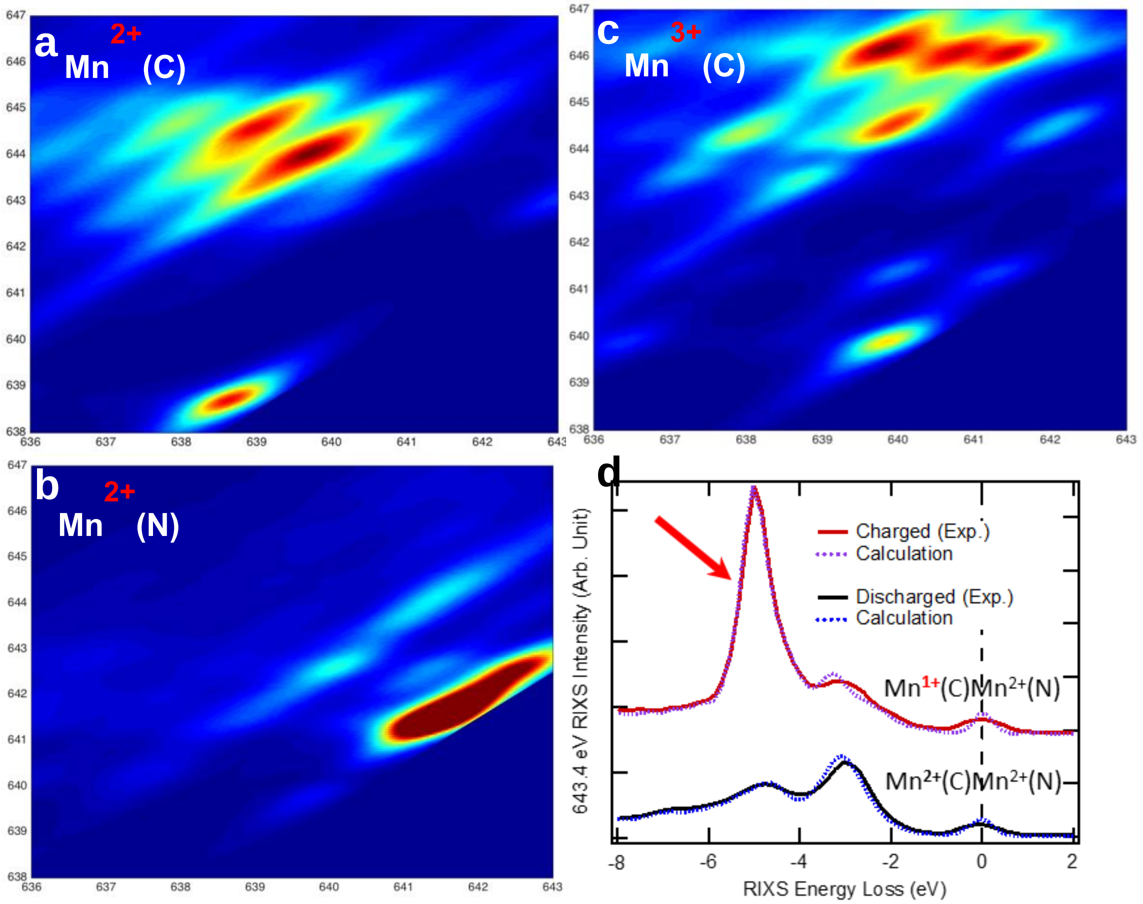

Supplementary Figure 15 Theoretical calculations of RIXS maps of Mn-L_3 edge at different valence and coordination sites. (a) RIXS map of C-coordinated Mn^{2+}. (b) RIXS map of N-coordinated Mn^{2+}. (c) RIXS map of C-coordinated Mn^{3+}. Only Mn^{1+}(C) shows the much enhanced d-d excitation feature at 643.4 eV excitation and 638.4 eV emission energies as observed in experiments (Figure 4). (d) shows the perfect agreement between the RIXS experimental cut (solid lines, ±0.5 eV range) and theoretical calculations (dotted lines) with the specified Mn state configuration as marked on the figure. Red arrow indicates the fingerprinting feature of Mn^{1+}(C) at 643.4 eV excitation energy.

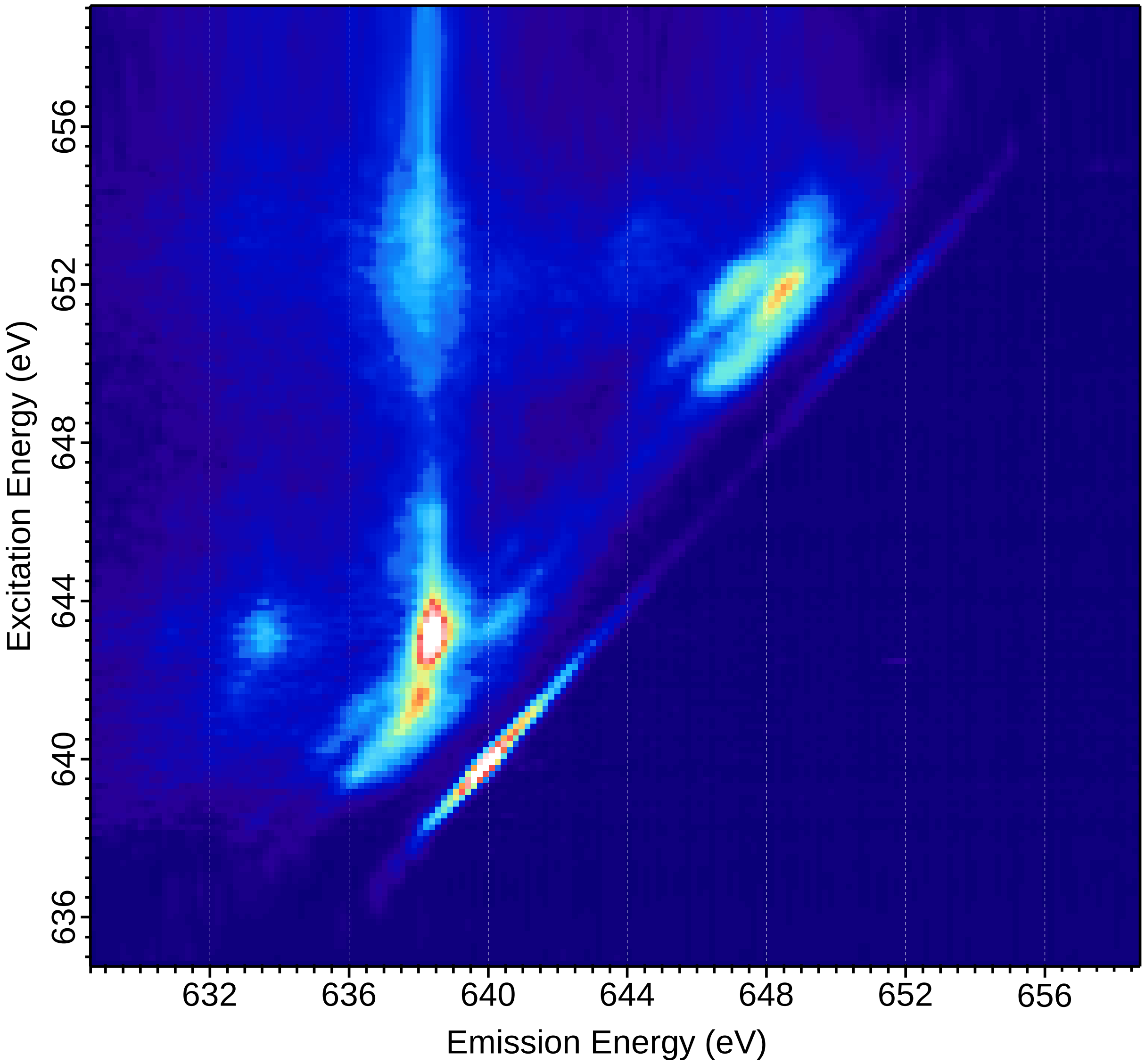

Supplementary Figure 16 Full energy range Mn-L RIXS map collected on MnHCMn electrodes at charged state (Mn^{1+}). The two packets of intensity around 642 and 652 eV excitation energies represent the RIXS features of Mn-L_3 and Mn-L_2 edges. The “vertical feature” along 538 eV emission energy represents the band-like fluorescence signal, indicating non-localized states in the system. Detailed analysis and further theoretical calculations of the RIXS results will be available in separated works.

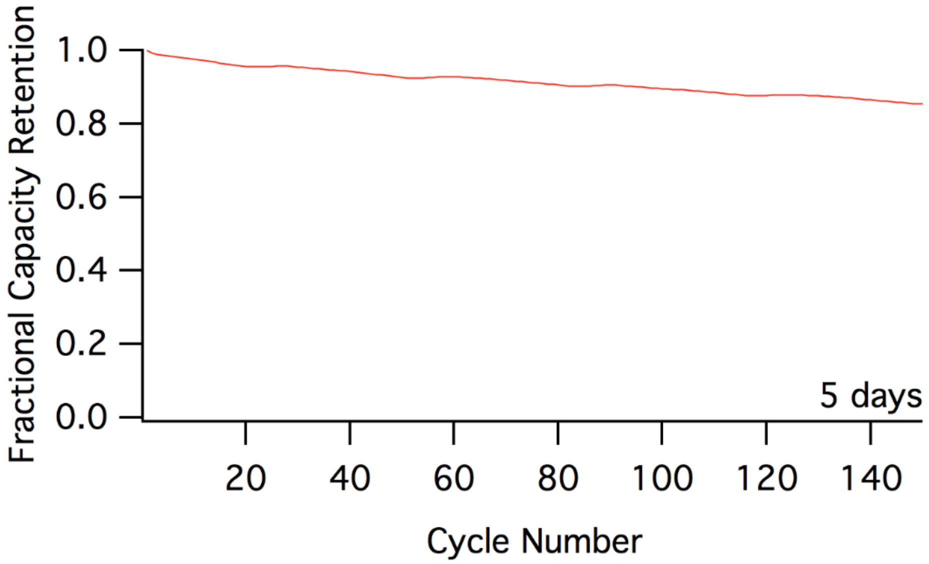

Supplementary Figure 17 Typical capacity retention of CuHCF electrode cycled in aqueous electrolyte with 1 M $NaClO_4$. There is a 15% capacity loss after 5 days of 1C, 100% DOD cycling.

Supplementary Tables

Supplementary Table 1. The calculated *d* electron orbital occupancies for Mn^{II}(C) and Mn^{I}(C), at 0% (1D) and 100% (1Ch) state of charge, respectively.

	d **electron occupancy**		
	t_{2g}	e_g	total
**Mn^{2+}(C)**	4.5223	0.1354	4.658
**Mn^{1+}(C)**	5.4387	0.0913	5.530
**Diff.**	0.9164	-0.0441	0.872

Supplementary Table 2 CuHCF ($Na_{0.67}Cu[Fe(CN)_6]_{0.67}\cdot 3.8H_2O$) cathode cost.

Precursor	Precursor $ kg^{-1}	Precursor active mass fraction	Precursor active $ kg^{-1}	Precursor mass fraction in CuHCF	Precursor $ kg^{-1} in CuHCF
$CuSO_4\cdot 5H_2O$	2.00	0.254 (Cu)	7.85	0.22	1.73
$Na_4Fe(CN)_6\cdot 10H_2O$	1.30	0.485 $(NaFe(CN)_6)_{0.67}$	2.68	0.54	1.45
Water	0	1	0	0.24	0
Total				1.00	**3.19**

Supplementary Table 3 MnHCMn ($Na_{1.24}Mn[Mn(CN)_6]_{0.81}\cdot 2.1H_2O$) anode cost.

Precursor	Precursor \$ kg^{-1}	Precursor active mass fraction	Precursor active \$ kg^{-1}	Precursor mass fraction in MnHCMn	Precursor \$ kg^{-1} in MnHCMn
$Mn(OAc)_2$	2.00	0.312 (Mn)	5.13	0.33	1.67
NaCN	1.30	0.629 ($Na_{0.21}CN$)	1.59	0.55	0.87
Water	0	1	0	0.13	0
Total				1.00	**2.54**

Supplementary Notes

Supplementary Note 1 Battery Full-cell Cost Estimation Summary

Publicly available bulk quotes for electrode and electrolyte precursors result in an estimated bill of materials of $68/kWh for the active materials (anode, cathode, electrolyte) in the cell described here. Assuming the same 50% manufacturing cost for PBA cells as for Li-ion cells, this results in a cost of goods sold of about $140/kWh, lower than the projected cost floor of Li-ion cells[2]. For PBA cells, manufacturing costs may be a smaller fraction of the total cell cost than for Li-ion cells. As PBA materials are hydrates and the electrolyte contains water, cell assembly does not require a dry room, one of the largest capital expenses in Li-ion manufacturing (see P.A. Nelson et al., Modeling the Performance and Cost of Lithium-Ion Batteries for Electric-Drive Vehicles. 2011). With further optimization, we could assume a full cell cycle life of ~10,000 cycles demonstrated for the PBA electrodes. With a conservative $200/kWh sale price, 95% energy utilization and 97% energy efficiency (representing no further performance optimization), a cell-level LCOE is estimated as $0.02/kWh·cycle. This LCOE surpasses the most aggressive US Department of Energy target (see US Department of Energy ARPA-E FOA document 2010). In comparison, the LCOE of natural gas turbines currently used for ramping of volatile renewables is about $0.08/kWh·cycle, while the LCOEs of other battery technologies are even higher[3]. Further optimization of PBA cell lifetime and cost will result in an even lower LCOE. Thus, Na-ion battery systems based on the PBA electrodes will dramatically decrease the cost for grid-scale energy storage.

Note 2 Battery Cost Calculation Methods

For the PBA cell chemistry, the costs of the active materials were calculated based on publicly available internet quotes for reagent grade chemical precursors gathered in early 2015. The one

change in precursor from those used in cells described here is the substitution of sodium ferrocyanide for potassium ferricyanide as a precursor for the CuHCF cathode, which allows it to start at full discharge, thus eliminating the electrochemical pre-reduction process used here.

The cost of PBA based cathode and anode is analyzed in Supplementary Table 2 and Table 3, respectively. The cost of our co-solvent electrolyte and the total cost of the reported full cell are analyzed below.

1 M $NaClO_4$, 90% MeCN (volume), 10% water electrolyte cost:

- 1 M $NaClO_4 \cdot H_2O$ ($1.00/kg, 2.02 kg/L) = $0.14 $NaClO_4$/L electrolyte and 1.8% vol. H_2O
- Neglect the cost of the additional 8% vol. H_2O as negligible.
- 99% MeCN ($2.00/kg, density 0.786 kg/L) = $1.41/L electrolyte.
- Total electrolyte cost = $1.59/L = $1.64/kg

Calculation of the PBA cell materials cost per energy:

- Cathode: $3.19/kg, 62 Ah/kg
- Anode: $2.54/kg, 66 Ah/kg

For 1 kWh of 1.55 V average voltage cells, a capacity of 1000/1.55 = 645 Ah is required.

- Cathode: 645 Ah/(62 Ah/kg) = 10.40 kg = $33.18
- Anode: 645 Ah/(66 Ah/kg) = 9.78 kg = $24.84

Assume that the composite electrodes have a bulk materials density of 2 kg/L (typical for PBAs, carbon, and binders), and a porosity of 33%. Then, 0.33 L = 0.32 kg electrolyte per kg electrode is required. Then, for the 20.18 kg of total electrode mass, 6.45 kg electrolyte is required at a cost of $10.59.

Total active materials costs per kWh:

$33.18 (cathode) + $24.84 (anode) + $10.59 (electrolyte) = $68.61

Supplementary References

1. Eisebitt, S., Böske, T., Rubensson, J. E. & Eberhardt, W. Determination of absorption coefficients for concentrated samples by fluorescence detection. *Phy. Rev. B* **47**, 14103 (1993).
2. Brodd, R. J. & Helou, C. Cost comparison of producing high-performance Li-ion batteries in the U.S. and in China. *J. Power Sources* **231**, 293–300 (2013).
3. Yang, Z. et al. Electrochemical energy storage for green grid. *Chem. Rev.* **111**, 3577–3613 (2011).
